# Selective breeding modifies *mef2ca* mutant incomplete penetrance by tuning the opposing Notch pathway

**DOI:** 10.1371/journal.pgen.1008507

**Published:** 2019-12-02

**Authors:** Juliana Sucharov, Kuval Ray, Elliott P. Brooks, James T. Nichols

**Affiliations:** Department of Craniofacial Biology, School of Dental Medicine, University of Colorado Anschutz Medical Campus, Aurora, Colorado, United States of America; UC-Berkeley, UNITED STATES

## Abstract

Deleterious genetic mutations allow developmental biologists to understand how genes control development. However, not all loss of function genetic mutants develop phenotypic changes. Many deleterious mutations only produce a phenotype in a subset of mutant individuals, a phenomenon known as incomplete penetrance. Incomplete penetrance can confound analyses of gene function and our understanding of this widespread phenomenon remains inadequate. To better understand what controls penetrance, we capitalized on the zebrafish *mef2ca* mutant which produces craniofacial phenotypes with variable penetrance. Starting with a characterized *mef2ca* loss of function mutant allele, we used classical selective breeding methods to generate zebrafish strains in which mutant-associated phenotypes consistently appear with low or high penetrance. Strikingly, our selective breeding for low penetrance converted the *mef2ca* mutant allele behavior from homozygous lethal to homozygous viable. Meanwhile, selective breeding for high penetrance converted the *mef2ca* mutant allele from fully recessive to partially dominant. Comparing the selectively-bred low- and high-penetrance strains revealed that the strains initially respond similarly to the mutation, but then gene expression differences between strains emerge during development. Thus, altered temporal genetic circuitry can manifest through selective pressure to modify mutant penetrance. Specifically, we demonstrate differences in Notch signaling between strains, and further show that experimental manipulation of the Notch pathway phenocopies penetrance changes occurring through selective breeding. This study provides evidence that penetrance is inherited as a liability-threshold trait. Our finding that vertebrate animals can overcome a deleterious mutation by tuning genetic circuitry complements other reported mechanisms of overcoming deleterious mutations such as transcriptional adaptation of compensatory genes, alternative mRNA splicing, and maternal deposition of wild-type transcripts, which are not observed in our system. The selective breeding approach and the resultant genetic circuitry change we uncovered advances and expands our current understanding of genetic and developmental resilience.

## Author summary

Some deleterious gene mutations only affect a subset of genetically mutant animals. This widespread phenomenon, known as mutant incomplete penetrance, complicates discovery of causative gene mutations in both model organisms and human disease. This study utilized the zebrafish *mef2ca* transcription factor mutant that produces craniofacial skeleton defects with incomplete penetrance. Selectively breeding zebrafish families for low- or high-penetrance mutants for many generations created different zebrafish strains with consistently low or high penetrance. Comparing these strains allowed us to gain insight into the mechanisms that control penetrance. Specifically, genes under the control of *mef2ca* are initially similarly expressed between the two strains, but differences between strains emerge during development. We found that genetic manipulation of these downstream genes mimics the effects of our selective breeding. Thus, selective breeding for penetrance can change the genetic circuitry downstream of the mutated gene. We propose that small differences in gene circuitry between individuals is one mechanism underlying susceptibility or resilience to genetic mutations.

## Introduction

### Some mutant organisms do not manifest a phenotype

Certain gene mutations arising from traditional zebrafish forward-genetic screens only produce a phenotype in a subset of mutant individuals, a phenomenon known as incomplete penetrance [1]. Incomplete penetrance has long been appreciated in many organisms, although the mechanisms underlying the phenomenon are not completely clear. How animals might overcome a deleterious mutation is a long-standing question of considerable interest to developmental geneticists.

Advances in next-generation sequencing technology have dramatically reduced the cost of whole-genome sequencing. As a result, new efforts are underway to sequence genomes from healthy humans in addition to genomes from disease-affected individuals [2]. Surprisingly, a recent sequencing study uncovered human individuals harboring mutations for severe Mendelian conditions, thought to be fully penetrant, that do not display a disease phenotype [3]. Thus, incomplete penetrance among human genetic diseases might be more widespread than previously appreciated. The discovery of healthy individuals buffering the effects of deleterious mutations led to the emerging concept of genetic resilience, or the ability of an organism to overcome a deleterious mutation. Model systems like the zebrafish provide an opportunity to test mechanistic hypotheses about genetic resilience.

### Various reported mechanisms underlie mutants without a phenotype

The rapid production of zebrafish reverse-genetic mutants in recent years has revealed that predicted loss of function mutations in many genes do not produce overt phenotypic changes [4]. Mechanisms proposed to underlie zebrafish reverse genetic mutants that do not manifest a phenotype include genetic compensation [5] and alternative mRNA processing to omit mutation-containing exons [6]. Maternally contributed wild-type transcripts can also mask zygotic mutant phenotypes [7].

Studies in mice have established that genetic background affects penetrance [8–11]. Genetic background is a catch-all term for general genomic differences, and therefore we know little about the specific mechanisms that modify penetrance in different backgrounds. Additionally, the reason why some backgrounds are more effective than others at overcoming particular mutations is not well understood.

Proposed incomplete penetrance mechanisms of human disease-causing alleles include age, sex, environment, and allele type [12]. However, these mechanisms cannot fully account for incomplete penetrance, as model organism studies often account for these variables and the phenomenon persists [13].

Discrete second-site mutations can affect penetrance, and secondary screens in genetic models have uncovered genes that can modify mutant phenotypes [14–16]. Moreover, spontaneous secondary genetic mutations can arise in response to normal laboratory culture of mutant yeast, suggesting genomic imbalance can drive evolution of rescuing mutations without deliberate selection [17]. Thus, genetic network rewiring might occur following generations of selection and mutant organisms might be especially prone to adaptive variations in gene networks. In fact, recent work demonstrates that small changes in gene expression can have large phenotypic consequences in a mutant context whereas the same small changes in gene expression do not alter the wild-type phenotype [18]. Therefore, changes in gene expression that alter mutant phenotypes can emerge following selective pressure without deleterious consequences in wild types or heterozygotes.

In some systems, incomplete penetrance can be ascribed to stochastic variation in gene expression [19]. However, we have previously shown that penetrance is heritable and can be driven downward or upward by selective breeding, demonstrating that incomplete penetrance is not always due to stochasticity [20]. There have been few examples of zebrafish selective breeding to alter phenotypes described. However, one group selecting for body size differences demonstrated that transcriptional changes arise following selection [21]. Therefore, we wished to determine if transcriptional changes occurred following selective breeding for low- and high-penetrance in our system.

### Mutations in the *myocyte enhancer factor 2c* transcription factor encoding gene exhibit craniofacial defects, with incomplete penetrance

The *myocyte enhancer factor 2c* (*Mef2c*) transcription factor encoding gene is a highly conserved, well-studied developmental gene present in metazoans. Mutations in *MEF2C*/*Mef2c*/*mef2ca* produce craniofacial defects in humans, mice and zebrafish [22–24]. In humans, *MEF2C* mutant patients display highly variable and incompletely penetrant craniofacial phenotypes in derivatives of the first and second branchial arches. At least one human with a mutation in *MEF2C* exhibits an extremely mild phenotype, suggesting humans can be naturally resilient to mutations in *MEF2C* [25].

Like humans, zebrafish *mef2ca* mutants also display highly variable and incompletely penetrant craniofacial phenotypes in derivatives of the first and second branchial arches. The zebrafish *mef2ca*^*b1086*^ allele we utilize in this study encodes a premature termination codon immediately following the MADS box, which encodes the DNA binding domain [24]. We previously demonstrated that the opercle bone, which supports the gill cover, has particularly variable phenotypes in these *mef2ca* mutants [26]. Penetrance of this variable phenotype, ectopic bone near the opercle, is heritable and subject to manipulation by selective breeding [20]. It is still unknown whether selective breeding for just the ectopic bone phenotype affects other *mef2ca* mutant phenotypes in our system. Moreover, how the *mef2ca* genetic circuit might change in response to selection for low or high penetrance remains to be determined.

### *mef2ca* functions in the Endothelin pathway, which opposes the Jagged/Notch signaling axis during craniofacial development

Vertebrate *Mef2c* functions as a downstream effector of Endothelin (Edn1) signaling to pattern dorsoventral identity in neural crest cells (NCCs) in jawed vertebrates [23, 24, 27, 28]. Edn1 signaling is opposed by Jagged/Notch (Jag/N) signaling during craniofacial patterning and loss of function of the Notch ligand *jag1b* can rescue an *edn1* loss of function mutation [29, 30]. Even loss of a single copy of *jag1b* rescues *edn1* mutant phenotypes, emphasizing the delicate balance of these two opposing signaling pathways [29]. In situ hybridization gene expression studies indicate that Edn1 signaling and Jag/N signaling act oppositely on at least one shared transcriptional target; *dlx5a* expression is activated by Edn1 [31] and repressed by Jag/N [29]. However, subtle gene expression differences that might underlie differential penetrance are not likely to be detected by in situ hybridization. More sensitive measurements of gene expression are required to uncover small, but functionally important, differences.

Studies in mammalian cultured cells demonstrate that activated Notch can directly bind to MEF2C, blocking its DNA binding and transcriptional activity [32, 33]. These findings motivate the hypothesis that Mef2c links the Edn1 and Jag/N signaling pathways, perhaps functioning as a shared node mediating crosstalk between these oppositional pathways. Although activated forms of Notch and overexpression alleles of *Mef2* synergize in *Drosophila* [34], Notch pathway genes and *Mef2c* have not been tested for genetic interactions in vertebrates. Moreover, the effect of Mef2c on Notch signaling has not yet been explored.

Here, we capitalize on our selective breeding paradigm for *mef2ca* low and high penetrance to advance our understanding of inheritance and developmental genetic mechanisms of incomplete penetrance. First, we discover that selecting just for penetrance of the ectopic bone phenotype produces penetrance changes in other *mef2ca*-associated phenotypes. These findings provide new support for a liability-threshold model of penetrance inheritance. Second, we report new extremes of phenotypic variation that can arise through selective breeding. At one extreme, breeding for low penetrance converts the *mef2ca* mutant allele from homozygous lethal to homozygous viable. At the other extreme, breeding for high penetrance converts the *mef2ca* mutant allele from recessive to dominant. Third, while we do not observe evidence for paralogous compensation, alternative splicing, or maternally deposited transcripts contributing to low penetrance, we do observe adaptive changes in the craniofacial patterning transcriptional network between low- and high-penetrance strains. Specifically, we provide evidence that the Jag/N pathway opposes *mef2ca* and that a specific node in the opposing Jag/N signaling network is disabled in the low-penetrance strain. Our gene expression and functional studies support a model in which selective breeding tunes the opposing Jag/N pathway affecting *mef2ca* mutant penetrance. These studies contribute to our understanding of how penetrance is inherited, how selective breeding for penetrance can produce either complete resilience or severe sensitivity to a deleterious mutation, and demonstrate one penetrance modifier mechanism that emerges in response to selective pressure.

## Results

### Long-term selective breeding modifies *mef2ca* mutant ectopic bone penetrance

**Fig 1:**
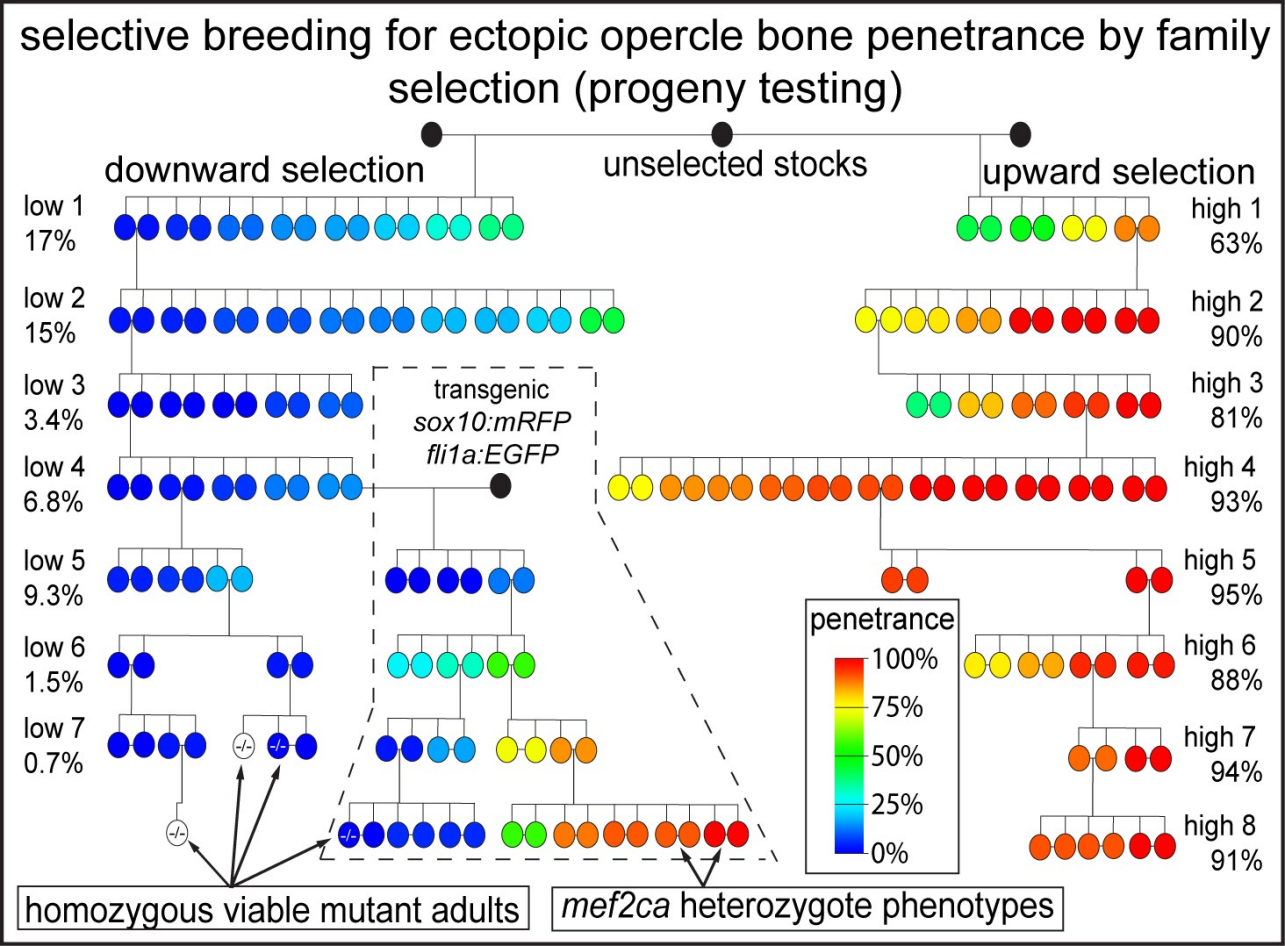
Selective breeding drives ectopic bone penetrance downward and upward. Full-sibling heterozygous *mef2ca* pairs were intercrossed and their homozygous mutant offspring were scored for penetrance of ectopic bone near the opercle. Mutant progeny penetrance scores were assigned to each breeding pair. Parents are color coded for offspring penetrance on this pedigree. Heterozygous offspring from selected pairs were raised for the next round of selection. A single low-penetrance individual from generation four was crossed to a double-transgenic *mef2ca* wild-type unselected individual (black circle). Indicated individuals from recent generations in the low-penetrance line are viable as *mef2ca* homozygous mutants. Indicated pairs from recent generations in the high-penetrance strain produce heterozygous offspring with *mef2ca*-associated phenotypes. Uncolored circles represent homozygous mutant adults that were recovered but were not tested for mutant offspring ectopic bone phenotype penetrance.

Zebrafish notoriously suffer from inbreeding depression [35]. However, we and others have demonstrated that through careful husbandry, full-sibling inbreeding is possible for multiple generations [36, 37]. Selective full-sibling inbreeding demonstrates that penetrance of ectopic bone near the opercle in *mef2ca* mutants is heritable [20]. We found that selective breeding by progeny selection can drive ectopic bone penetrance downward or upward over the course of three and four generations, respectively. Since that report, we continued our long term full-sibling selective breeding paradigm. Seven generations of full-sibling inbreeding for low penetrance has brought the ectopic bone penetrance to under 1%. In contrast, after eight generations of full-sibling inbreeding for high penetrance, ectopic bone penetrance is over 90% (Fig 1). The natural penetrance modifiers we selected for do not produce overt phenotypes when *mef2ca* is fully functional; genetic wild-type skeletons from both strains are indistinguishable from our laboratory wild-type AB strain at the current generation of selective breeding. To test if we might be able to re-derive low- and high-penetrance strains following the introduction of a non-selected genome, we outcrossed an individual animal from the fourth low-penetrance generation to a transgenic, but otherwise wild-type, individual and implemented selective breeding anew. The first generation with a ‘half-low’ background, a background arising from a selectively bred low-penetrance individual outcrossed to an unselected individual, exhibited low penetrance. However, we found that we could rapidly rederive a new high-penetrance strain by selective breeding (Fig 1). These data indicate that half backgrounds might be useful for introducing new genomic features while maintaining penetrance and emphasize the plasticity of penetrance in our system suggesting that relatively few loci shape penetrance.

### Selective breeding just for ectopic bone penetrance shapes other *mef2ca* mutant-associated phenotypes

**Fig 2:**
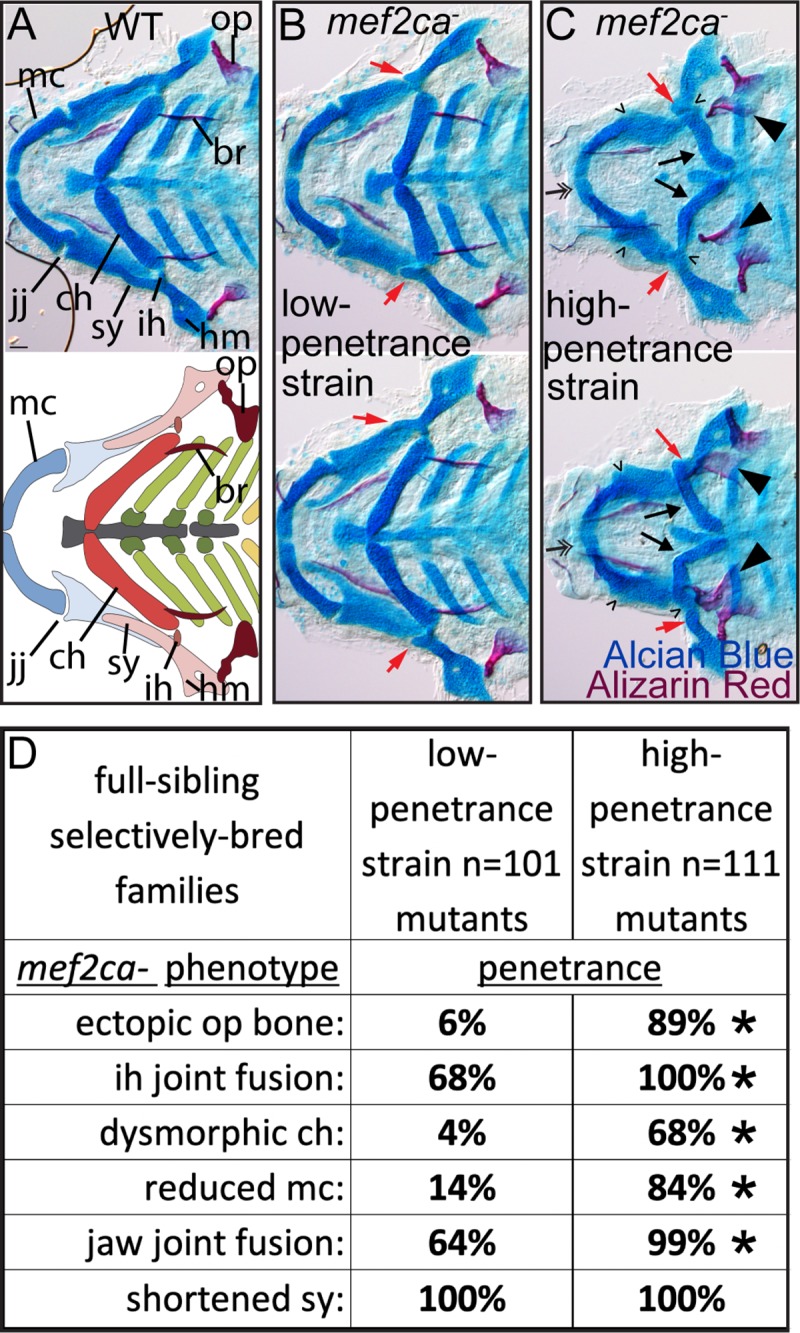
Selective breeding just for ectopic bone penetrance modifies penetrance of other *mef2ca*-associated craniofacial phenotypes to different extents. (A) Wild-type 6 days post fertilization (dpf) zebrafish larvae were stained with Alcian Blue and Alizarin Red to label cartilage and bone then flat mounted and imaged. The following craniofacial skeletal elements are indicated in the micrograph and graphic: opercle bone (op), branchiostegal ray (br), Meckel’s (mc), ceratohyal (ch), symplectic (sy) and hyomandibular (hm) cartilages, interhyal (ih) and jaw (jj) joints. Scale bar: 50 μm. (B, C) Penetrance of most phenotypes associated with *mef2ca* are reduced in homozygous mutants from the low-penetrance strain compared to homozygous mutants from the high-penetrance strain including: ectopic bone (arrowheads), interhyal- and jaw-joint fusions (^), dysmorphic ch (arrows), reduced mc (double arrowhead). A shortened sy cartilage (red arrows) remains fully penetrant in both strains. (D) Penetrance of *mef2ca*-associated phenotypes from full-sibling low- and high-penetrance families. Asterisks indicate statistically significant penetrance differences between homozygous mutants in low-penetrance compared with high-penetrance strains (Fisher’s exact test at alpha = 0.05).

In our selective breeding study, we bred for low and high penetrance of ectopic bone. During selection, we did not consider other *mef2ca*-associated phenotypes [24]; we neither purposefully selected for or against these other phenotypes. To determine if the other craniofacial phenotypes arising in *mef2ca* mutants are also sensitive to the modifiers that emerged through selective breeding for ectopic bone penetrance, we scored all phenotypes in Alcian Blue/Alizarin Red stained skeletal preparations from *mef2ca* mutant animals from the low- and high-penetrance strains (Fig 2A–2C). We observed that penetrance of most phenotypes in the cartilage skeleton were affected by selective breeding just for ectopic bone (Fig 2D). Interestingly, distinct skeletal phenotypes appeared differentially sensitive to the modifiers in the low- and high-penetrance strains. For example, in the low-penetrance strain penetrance of a dysmorphic ceratohyal and Meckel’s cartilages were considerably reduced, whereas the interhyal and jaw-joint fusions were only modestly reduced in the low-penetrance line. Meanwhile, the shortened symplectic cartilage phenotype remained fully penetrant in low-penetrance strain mutants.

**Fig 3:**
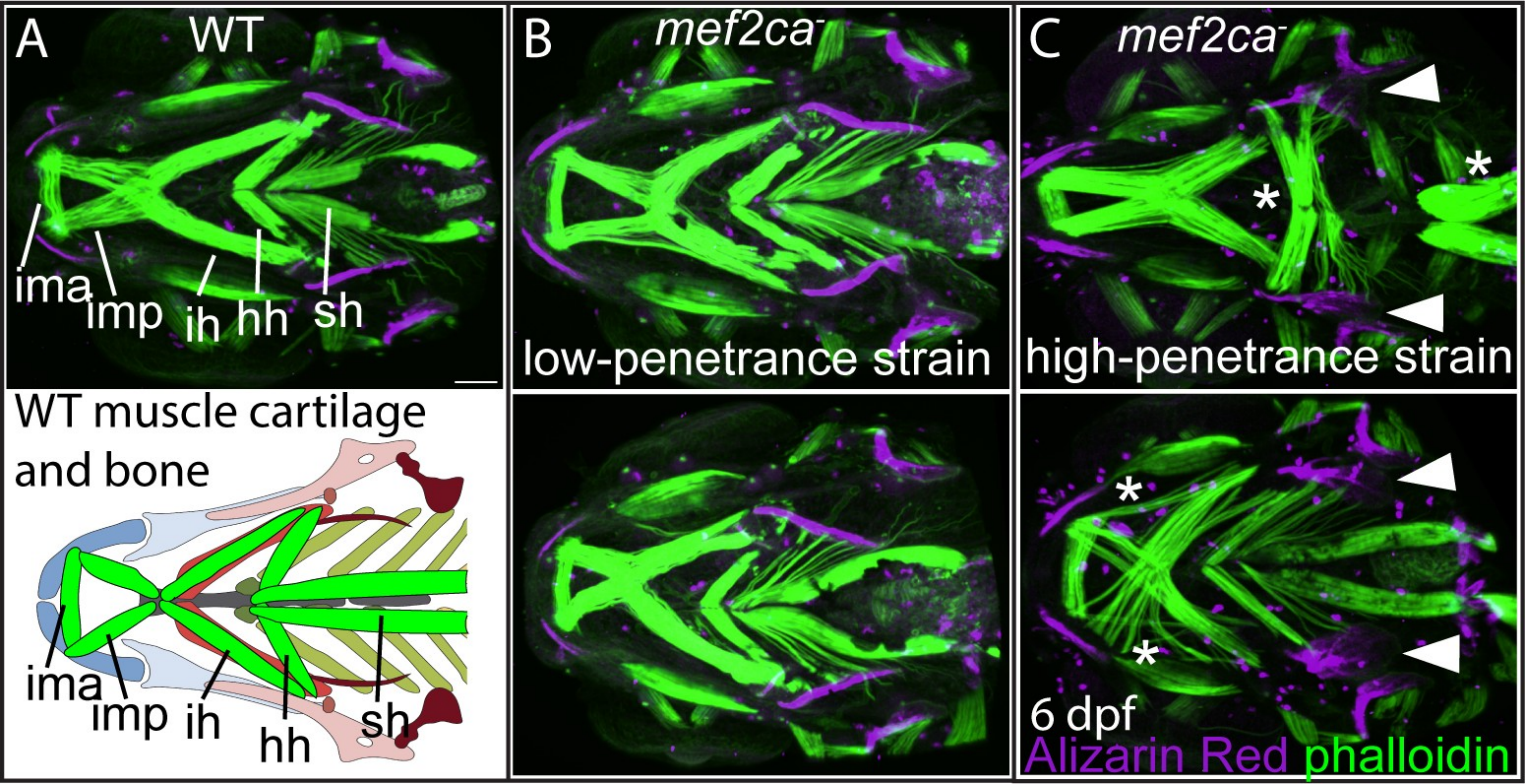
Selective breeding just for ectopic bone penetrance modifies penetrance of craniofacial muscle phenotypes. 6 dpf larvae were stained with Alizarin Red (magenta) and fluorescently labelled phalloidin (green) to mark bones and muscles, respectively. (A) The following craniofacial muscles are indicated in the micrograph and graphic: intermandibularis ant. (ima), intermandibularis post. (imp), interhyal (ih), hyohyal (hh), sternohyoideus (sh). Scale bar: 50 μm. (B) In the low-penetrance strain, *mef2ca* mutant muscles and bones resemble wild types. (C) In the high-penetrance strain, *mef2ca* mutants manifest muscle phenotypes (asterisks) in conjunction with ectopic bone phenotypes (arrowheads).

The chondrocytes and the osteoblasts within the disrupted elements in *mef2ca* mutants all derive from the same pool of progenitor cells, the NCCs in pharyngeal arches one and two. We next asked if *mef2ca* mutant phenotypes in structures derived from cell types beyond NCC are also sensitive to the modifiers in our strains and examined the craniofacial muscles, which are derived from the craniofacial mesoderm [38–40]. Although *mef2ca* is expressed in the craniofacial muscle progenitors [24], the *mef2ca* mutant craniofacial muscle phenotype has not been described. We find that in low-penetrance mutants, the craniofacial muscles look largely wild type (Fig 3A and 3B). In contrast, in the high-penetrance strain the craniofacial muscles are severely affected by loss of *mef2ca* (Fig 3C). Specifically, the hyohyal can invert and the intermandibularis posterior and interhyal muscles can misarticulate. The intermandibularis posterior and interhyal sometimes link arch one and two derived structures or splay at their junctions with the craniofacial cartilage. These results indicate that the modifiers that emerged following selection for the ectopic bone also affect the craniofacial muscles. However, we have not ruled out that the muscle phenotypes are secondary to the cartilage and bone phenotypes. In fact, previous work proposes that mispatterned NCCs in Edn1 pathway mutants cause mispatterned craniofacial muscles [41, 42]. Nevertheless, selecting on just the ectopic bone phenotype affects all phenotypes associated with *mef2ca* examined thus far. These findings indicate that selection for penetrance of one phenotype can affect the penetrance of all mutant-associated phenotypes to different extents.

### In the low-penetrance strain, homozygous mutants can survive the formerly homozygous-lethal *mef2ca* mutant allele

**Fig 4:**
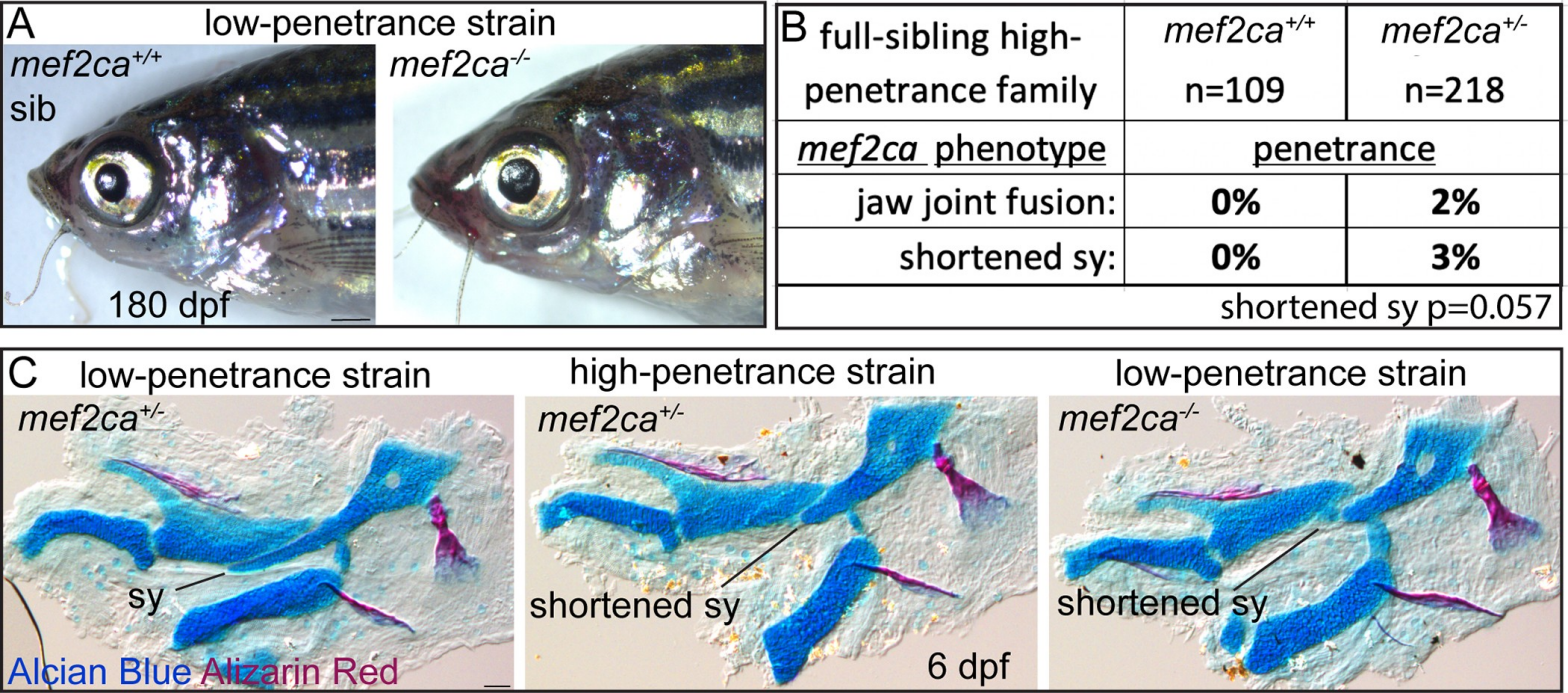
Selective breeding just for ectopic bone penetrance modifies the behavior of the *mef2ca*^*b1086*^ mutant allele. (A) Low-penetrance *mef2ca* heterozygous adults were intercrossed and offspring were grown to adulthood. Fin amputation and genotyping identified *mef2ca* homozygous adults, which were imaged alongside wild-type siblings. Scale bar: 1mm. (B) 6 dpf full-sibling individuals from a single high-penetrance family were stained with Alizarin Red (bone) and Alcian Blue (cartilage), then genotyped and *mef2ca* heterozygotes and homozygous wild types were scored for presence of all *mef2ca*-associated phenotypes. (C) 6 dpf Alizarin Red and Alcian Blue stained skeletons were dissected and flat mounted, lateral view. In these individuals, a shortened symplectic (sy) cartilage phenotype is evident in *mef2ca* heterozygotes from the high-penetrance strain and *mef2ca* homozygous mutants from the low-penetrance strain. Scale bar: 50 μm.

We have propagated *mef2ca* mutant alleles for over ten years and have never recovered a homozygous mutant adult in unselected strains. Our selection for low penetrance of ectopic bone rescued all the *mef2ca*-associated phenotypes, therefore we reasoned that rescuing these assorted phenotypes might render the animals able to survive the deleterious mutation and grow to adulthood. Remarkably, we now recover homozygous viable *mef2ca* mutant adults from the low-penetrance strain (Fig 1). We recover both male and female homozygous adults that are fertile and outwardly indistinguishable from their wild-type siblings (Fig 4A).

### In the high-penetrance strain, the formerly fully-recessive *mef2ca* mutant allele behaves as a partial dominant

Breeding for low penetrance buffered the strain to such an extent that homozygotes in the low-penetrance strain can survive the deleterious *mef2ca* mutation. We next hypothesized that, conversely, the high-penetrance strain animals might be so sensitive to *mef2ca* loss that phenotypes could appear in the heterozygous condition. Indeed, we are now recovering heterozygous animals with *mef2ca*-associated phenotypes (Fig 1). In this generation of selective breeding, we only observe the shortened symplectic and jaw-joint fusion phenotypes in *mef2ca* heterozygotes (Fig 4B and 4C). Consistently, these phenotypes are two of the most resistant to rescue in low-penetrance homozygous mutants (Fig 2D) and therefore likely the most sensitive to *mef2ca* loss. Notably, *mef2ca* heterozygotes from the high-penetrance strain resemble *mef2ca* homozygotes from the low-penetrance strain (Fig 4C).

### Neither transcriptional adaptation, alternative mRNA processing nor maternally deposited transcripts are likely to contribute to low penetrance in our system

**Fig 5:**
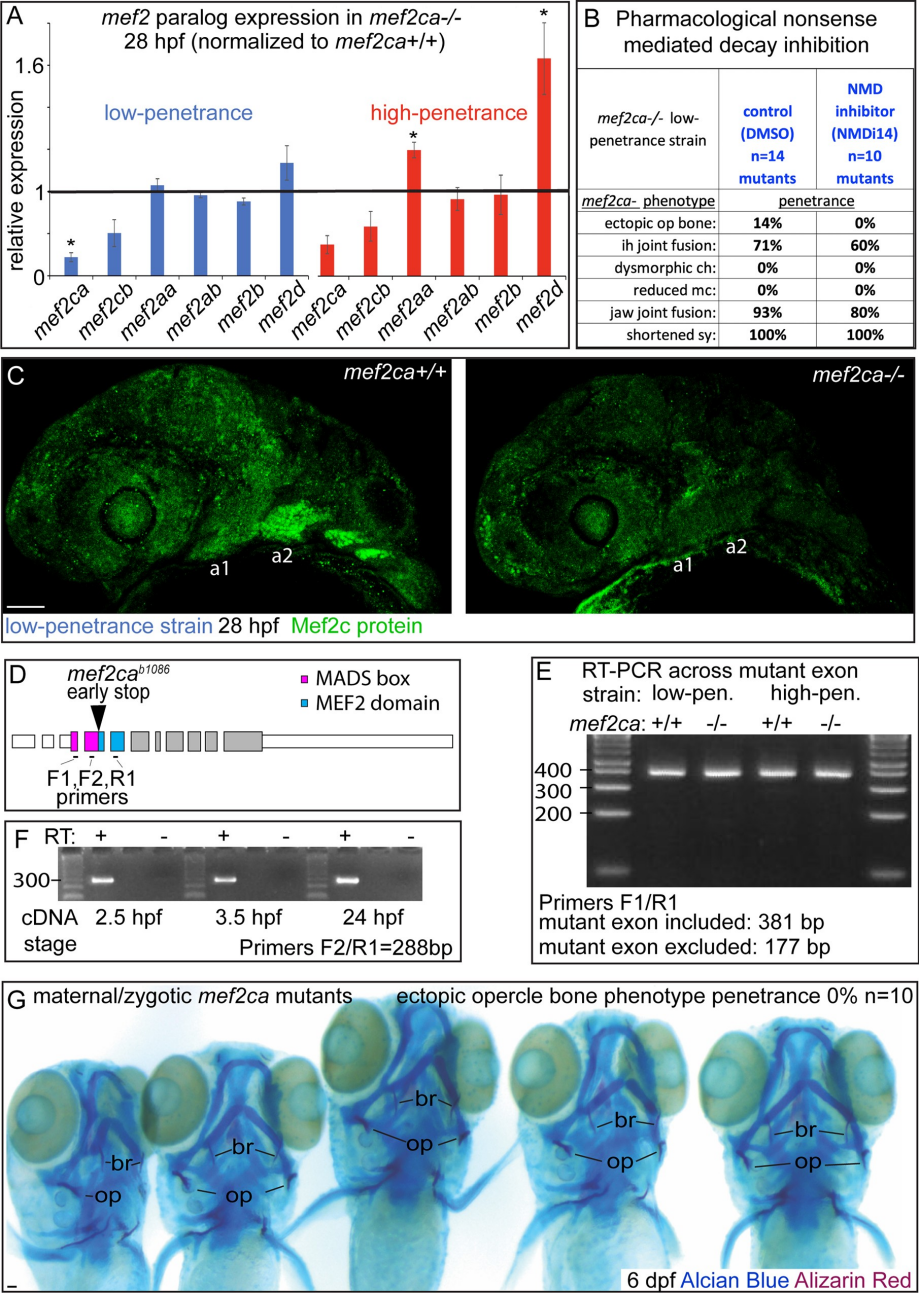
Transcriptional adaptation, alternative mRNA processing and maternally contributed transcripts do not contribute to strain-specific *mef2ca* mutant penetrance. (A) Head expression of all *mef2* paralogs was quantified by RT-qPCR in full-sibling *mef2ca* mutant and wild-type embryos from low- and high-penetrance strains at 28 hpf. Expression of each paralog in *mef2ca* mutants was normalized to wild-type sibling expression levels (black line). Asterisks mark paralogs with expression levels significantly different in *mef2ca* mutants compared with *mef2ca* wild types (T-test at alpha = 0.05). Error bars are standard deviation. (B) Animals heterozygous for *mef2ca* from the low-penetrance strain were intercrossed and offspring treated with nonsense-mediated decay inhibitor (NMDi14) or vehicle control (DMSO) from 18–42 hpf. Treated animals were fixed and stained with Alcian Blue (cartilage) and Alizarin Red (bone) at 6 dpf. Genotyped skeletal preparations were scored for penetrance of *mef2ca*-associated phenotypes. (C) Intercrosses from low-penetrance heterozygotes were immunostained with anti-MEF2C antibody. Genotyped animals were imaged in whole mount. 9/9 homozygous wild types had strong signal in the pharyngeal arches, 0/9 homozygous mutants exhibited any signal in the pharyngeal arches. Scale bar: 50 μm. (D) Exonic structure of *mef2ca* with protein domains and the location of the *mef2ca*^*b1086*^ mutation indicated. Primer pairs either span the mutant exon (F1/R1) or amplify across exon-exon boundary (F2/R1). (E) cDNA from wild-type and mutant siblings from low- and high-penetrance strains was used as a template for amplification across the mutant exon. These primers are predicted to amplify bands of indicated different sizes if the mutant exon is included versus excluded from mature transcripts. (F) cDNA from wild-type animals was collected before and after zygotic genome activation and used as a template for PCR amplification across an exon-exon junction. Reverse transcriptase (RT) negative control ensured that only cDNA and not genomic DNA was amplified under these conditions. (G) A homozygous mutant female was crossed with a heterozygous male sibling from the low-penetrance strain and offspring were fixed and stained with Alcian Blue (cartilage) Alizarin Red (bone) at 6 dpf. Genotyped maternal-zygotic mutants were scored for ectopic bone which develops between the opercle (op) and the branchiostegal ray (br) in mutants from the high-penetrance strain. Scale bar: 100 μm.

Deleterious mutations that induce nonsense-mediated decay can drive transcriptional upregulation of compensatory genes, often paralogs [43, 44]. This phenomenon, known as transcriptional adaptation, is seen in mouse *Mef2c* mutants where *Mef2b* expression is upregulated in embryonic hearts [45] motivating the hypothesis that paralog upregulation in *mef2ca* mutants contributes to low penetrance in our selectively-bred line. To determine if differences in *mef2* paralog upregulation might underlie heritable penetrance differences between our selectively-bred strains, we quantified expression of the six annotated *mef2* paralogs by RT-qPCR in wild-type and *mef2ca* mutant embryo heads from both the low- and high-penetrance strains. While we do detect decreased levels of *mef2ca* transcripts in *mef2ca* mutants at 28 hours post fertilization (hpf), likely due to nonsense-mediated decay which is expected for a premature termination codon allele like this one, we find no evidence for paralog upregulation by the deleterious *mef2ca* mutation in the low-penetrance strain (Fig 5A). Unexpectedly, the high-penetrance strain does show evidence of *mef2d* and *mef2aa* paralog upregulation in *mef2ca* mutants. Thus, while there may be some transcriptional adaptation by paralogs in the high-penetrance strain, this mechanism is not likely to contribute to heritable low-penetrance in our system. However, these experiments do not rule out that differences in paralog gene encoded proteins might account for strain-specific penetrance. Future study is required to determine if mutagenizing the paralogs might increase severity of the high-penetrance strain further still.

Nonsense-mediated decay might drive transcriptional adaptation of other pathway genes, not just *mef2ca* paralogs. Therefore, to test if nonsense-mediated decay is required for low penetrance in our selectively bred strain, we used the pharmacological inhibitor (NMDi14) as described in the report discovering transcriptional adaptation [43]. Consistent with our finding that no *mef2* paralogs are upregulated in *mef2ca* mutants in this strain, we do not observe any significant changes in *mef2ca* mutant phenotype penetrance following nonsense-mediated decay inhibition (Fig 5B).

It is possible that low-penetrance strain mutants might overcome the deleterious mutation by stop-codon read-through of the premature termination codon in *mef2ca*^*b1086*^ mutants [46]. To test if full-length functional Mef2ca protein might be produced in low-penetrance mutants, we immunostained homozygous wild types and *mef2ca* mutants from the low-penetrance strain with an antibody raised against full-length human MEF2C (Fig 5C). In homozygous wild types we observed a strong signal in the nuclei of neural crest cells in the pharyngeal arches. In contrast, Mef2ca protein is undetectable in the pharyngeal arches of low-penetrance homozygous mutants. These results indicate that stop-codon read-through producing functional protein is not likely to contribute to low penetrance in our system.

A recent report revealed that zebrafish can overcome some deleterious ENU and CRISPR/Cas9 lesions by alternative mRNA processing [6]. Since the mutated exon in the case of *mef2ca*^*b1086*^ contains much of the DNA binding domain encoding MADS box (Fig 5D), it seems unlikely that a transcript lacking this exon would be sufficient for *mef2ca* function. Nevertheless, to test if alternative mRNA processing is a mechanism of variable, heritable penetrance in our system, we performed PCR reactions on cDNA from wild-type and mutant embryos from both low- and high-penetrance strains. We found no evidence that the exon containing the deleterious lesion was alternatively processed in any condition (Fig 5E). Thus, alternative mRNA processing does not appear to contribute to *mef2ca* low penetrance in our system.

Maternally-loaded transcripts can contribute to development in the absence of functional zygotic genes, so we next hypothesized that differential deposition of maternal transcripts between strains might contribute to variable penetrance. In this scenario, heterozygous mothers might deposit high or low levels of wild-type *mef2ca* transcripts contributing to low or high penetrance of *mef2ca* phenotypes in zygotic mutants, respectively. We were able to detect maternally deposited *mef2ca* transcripts in embryos prior to zygotic genome activation, which occurs around 3 hpf, indicating that *mef2ca* is maternally deposited (Fig 5F). To test if maternally loaded wild-type *mef2ca* transcript is required for low penetrance, as our hypothesis predicted, we generated maternal-zygotic *mef2ca* mutants by crossing a low-penetrance homozygous mutant female to a heterozygous male. In this experiment, there will be no wild-type *mef2ca* RNA deposited by the mother to potentially rescue the offspring mutant phenotype. We find that maternal-zygotic mutants from this low-penetrance background give rise to low-penetrance offspring (Fig 5G). Thus, maternally deposited wild-type *mef2ca* mRNA is not required for low penetrance.

We conclude that neither paralogous compensation, stop-codon read-through, alternative splicing, nor maternally loaded wild-type transcripts are likely to contribute to heritable, variable *mef2ca* mutant penetrance.

### The *mef2ca* transcriptional target gene *dlx5a* shows strain-specific expression dynamics

**Fig 6:**
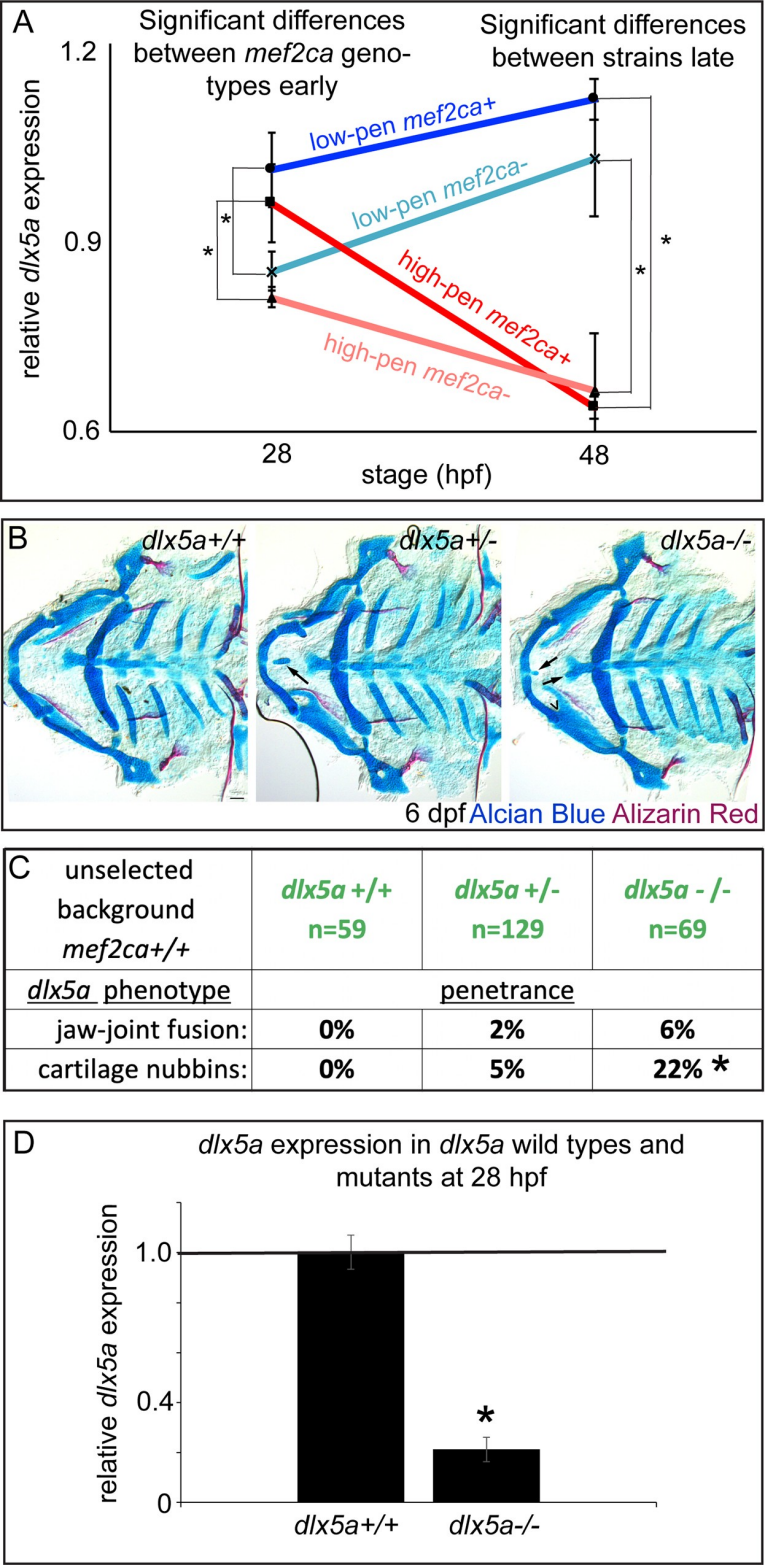
The *mef2ca* transcriptional target *dlx5a* is differentially regulated between low- and high-penetrance strains and is required for normal craniofacial development in unselected strains. (A) Head expression of *dlx5a* was quantified by RT-qPCR in full-sibling *mef2ca* mutant and wild-type embryos from low- and high-penetrance strains at 28 and 48 hpf. Expression levels for all conditions were normalized to *mef2ca* wild types at 28 hpf. Asterisks at 28 hpf mark significantly different expression levels between *mef2ca* mutants compared with *mef2ca* wild types within each strain. Asterisks at 48 hpf mark significantly different expression levels between low- and high-penetrance strains within each *mef2ca* genotype (T-test at alpha = 0.05), error bars are standard deviation. (B) Heterozygous *dlx5a*^*j1073Gt*^ adults from an unselected background were intercrossed, 6 dpf offspring were fixed and stained with Alcian Blue (cartilage) and Alizarin Red (bone). Stained skeletons were genotyped, and flat mounts were imaged. Arrows mark ectopic cartilage nubbins, caret indicates jaw-joint fusion. Scale bar: 50 μm. (C) Penetrance scores for phenotypes associated with *dlx5a* heterozygotes and mutants in an unselected background. Asterisk indicates significance at alpha = 0.05 Fisher’s exact test compared with *dlx5a+/+*. (D) Head expression of *dlx5a* was quantified by RT-qPCR in full-sibling *dlx5a* wild-type and mutant embryos at 28 hpf. Expression in *dlx5a* mutants was normalized to wild-type sibling expression levels (black line). Asterisk marks expression level significantly different in *dlx5a* mutants compared with *dlx5a* wild types (T-test at alpha = 0.05). Error bars are standard deviation.

We next hypothesized that changes to the *mef2ca* genetic network might contribute to heritable penetrance differences. To understand how the genetic circuit might differ between strains, we examined a canonical, likely direct, *mef2ca* transcriptional target. Expression of the transcription factor encoding gene *dlx5a* depends upon *mef2ca* function in both mouse and zebrafish [23, 24]. To quantitatively compare, with high sensitivity, how the expression of this known *mef2ca* transcriptional target might differ between wild types and mutants in both low- and high-penetrance strains, we measured *dlx5a* expression by RT-qPCR. We performed our assay during patterning of post-migratory NCCs in the pharyngeal arches, 28–48 hpf, when *mef2ca* is active (Fig 6A). At 28 hpf *dlx5a* expression was similarly significantly downregulated in *mef2ca* homozygous mutants in both strains compared with wild-type controls, suggesting that mutants from both strains are equally affected by the mutation at this stage. However, from 24–48 hpf, *dlx5a* expression in the low-penetrance strain increases in mutants and recovers to become indistinguishable between wild types and mutants. In contrast, in the high-penetrance strain *dlx5a* expression decreases in both wild types and mutants, where it also becomes indistinguishable between genotypes. Interestingly, comparing the same *mef2ca* genotype between strains at 48 hpf we find that *dlx5a* expression is significantly higher in the low-penetrance strain compared with the high-penetrance strain. Thus, *mef2ca* genotype primarily affects *dlx5a* expression early, while strain background is the cause of later differences. These results suggest that the functional differences between low- and high-penetrance strains may not be present early, but rather emerge during the course of development. There is a precedent for early losses in *dlx5a* to later recover in Edn1 pathway mutants. *furin1a* mutants are weak loss of Edn1 signaling and early losses in *dlx5a* expression recover during pharyngeal arch patterning [31], akin to what we observe for low-penetrance *mef2ca* mutants. In contrast, severe Edn1 pathway mutations like *edn1* itself do not recover early losses in *dlx5a* expression. This finding suggests that the differential ability of development to recover from early dysregulation might underlie differential penetrance.

### The loss of function *dlx5a*^*j1073Gt*^ allele shows heterozygous and homozygous mutant phenotypes in unselected strains

Strain-specific expression dynamics of the *mef2ca* target gene *dlx5a* motivate the hypothesis that *dlx5a* expression level functionally modifies *mef2ca* mutant penetrance. To test this hypothesis, we manipulated *dlx5a* function. The *dlx5a*^*j1073Gt*^ allele is a transgenic gene-trap insertion into the first exon of *dlx5a* widely used as a faithful transgenic reporter of *dlx5a* expression [26, 47–50]. The insertion is proposed to produce a loss of function allele associated with a shortened symplectic cartilage phenotype when homozygous [47]. In mice, *Dlx5* homozygous mutants produce dysmorphic craniofacial phenotypes including ectopic skeletal elements near Meckel’s cartilage directed towards the midline [51, 52]. We intercrossed *dlx5a*^*j1073Gt*^ heterozygotes from an unselected background and observed craniofacial phenotypes in both *dlx5a* heterozygotes and homozygotes (Fig 6B and 6C). Similar to the mouse, we observed ectopic cartilages near Meckel’s cartilage at the midline. We also observed jaw-joint fusions reminiscent of those in *mef2ca* mutants. We observe a significant downregulation of *dlx5a* transcript in *dlx5a*^*j1073Gt*^ homozygotes (Fig 6D) suggesting that *dlx5a*^*j1073Gt*^ is a loss of function allele and that *dlx5a* is haploinsufficient in this context. The phenotypes we observe in our experiments differ from those previously reported [47], suggesting that this allele might be sensitive to strain background differences.

### Genetically disrupting *dlx5a* function increases *mef2ca* mutant penetrance

**Fig 7:**
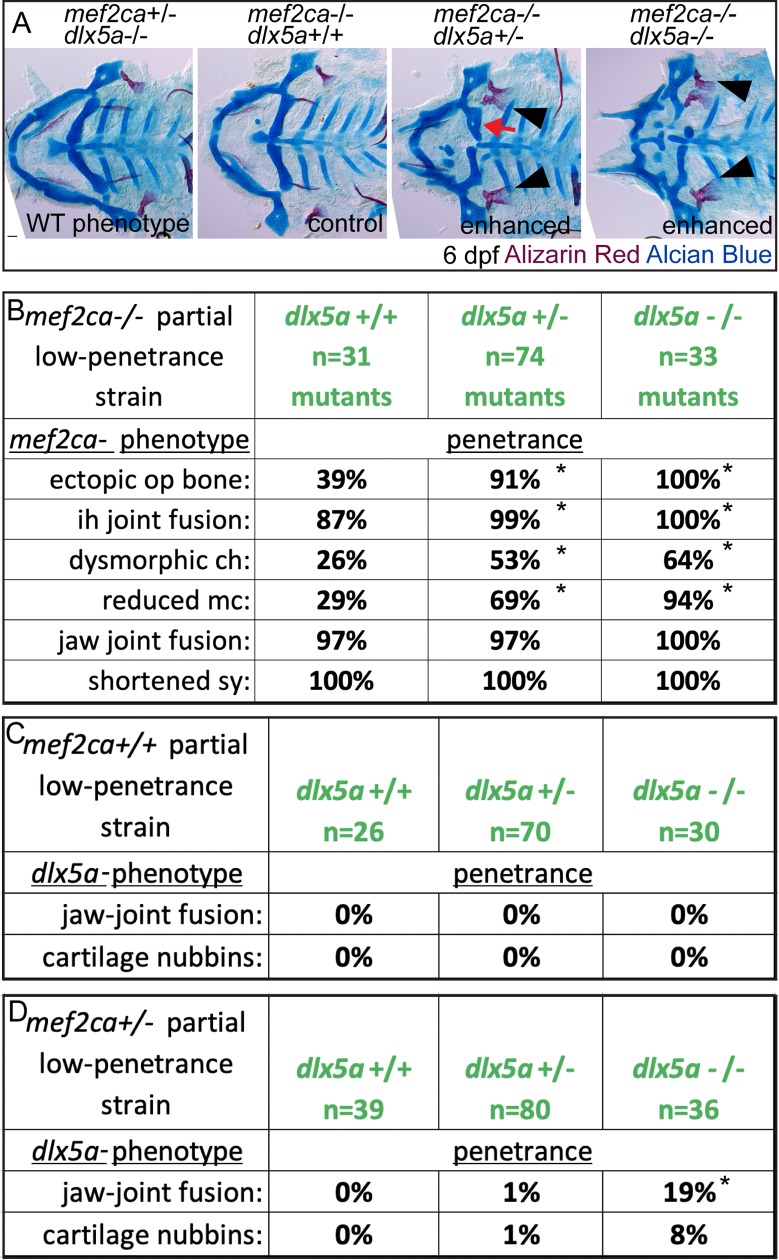
Loss of *dlx5a* function increases the penetrance of *mef2ca*-associated phenotypes. (A) Animals double heterozygous for *mef2ca* and *dlx5a* were intercrossed, raised to 6 dpf and fixed and stained with Alcian Blue (cartilage) and Alizarin Red (bone). Genotyped animals were flat mounted and imaged. In this family, *dlx5a* homozygous mutants appear wild type. But, removing functional copies of *dlx5a* increases *mef2ca* mutant penetrance. This includes phenotypes that we interpret to be ventral-to-dorsal transformations: br-to-op transformation (arrowheads), and ch-to-hm transformation (red arrow). Scale bar: 50 μm. (B) Genotyped *mef2ca;dlx5a* skeletal preparations were scored for penetrance of *mef2ca*-associated phenotypes. Asterisks indicate significance at alpha = 0.05 Fisher’s exact test compared with *dlx5a+/+*. (C, D) Genotyped *mef2ca;dlx5a* skeletal preparations were scored for penetrance of *dlx5a*-associated phenotypes.

Due to strain-specific *dlx5a* expression differences, we predicted that *dlx5a* might functionally participate in *mef2ca* mutant penetrance. To directly test this prediction, we disabled *dlx5a* in *mef2ca* mutants. We crossed animals heterozygous for the *dlx5a*^*j1073Gt*^ allele to individual low-penetrance *mef2ca* heterozygotes and reared offspring to adulthood resulting in *mef2ca;dlx5a* doubly heterozygous adults with a half-low penetrance background. As predicted, intercrossing these adults revealed that removing a single functional copy of *dlx5a* significantly increases penetrance of most *mef2ca* mutant-associated phenotypes, and that penetrance is further increased in doubly homozygous mutants (Fig 7A and 7B). Moreover, in some animals we observe nearly perfect ventral-to-dorsal homeotic transformations of the ceratohyal cartilage into a duplicated hyosymplectic cartilage complete with a foramen (Fig 7A, red arrow). These genetic interaction data suggest that *dlx5a* expression changes functionally shape *mef2ca* mutant penetrance, and that changes in the *mef2ca* genetic circuitry affecting *dlx5a* expression might underlie heritable penetrance variability.

Surprisingly, when the genetic interaction was tested in this half-low penetrance background, animals wild type for *mef2ca* and heterozygous or homozygous mutant for *dlx5a* were indistinguishable from genetic wild types (Fig 7C). This result contrasts with the *dlx5a* heterozygous and homozygous mutant phenotypes we observed in the unselected background (Fig 6). When *mef2ca* was heterozygous, *dlx5a*-associated phenotypes like jaw-joint fusions and ectopic cartilages were present in *dlx5a* heterozygotes and homozygous mutants (Fig 7D). Our finding that the *dlx5a* mutation produces phenotypes in *mef2ca* wild types from unselected strains, but not when introduced to the *mef2ca* low-penetrance strain, suggests that the modifiers in our selectively-bred strains are not specific to *mef2ca* but also modify phenotypes associated with other genes in the pathway. These data support the hypothesis that adaptive changes to the *mef2ca* genetic network underlie penetrance differences between our selectively-bred strains.

### Pharmacologically or genetically inhibiting Notch signaling decreases *mef2ca* mutant penetrance in the high-penetrance strain

**Fig 8:**
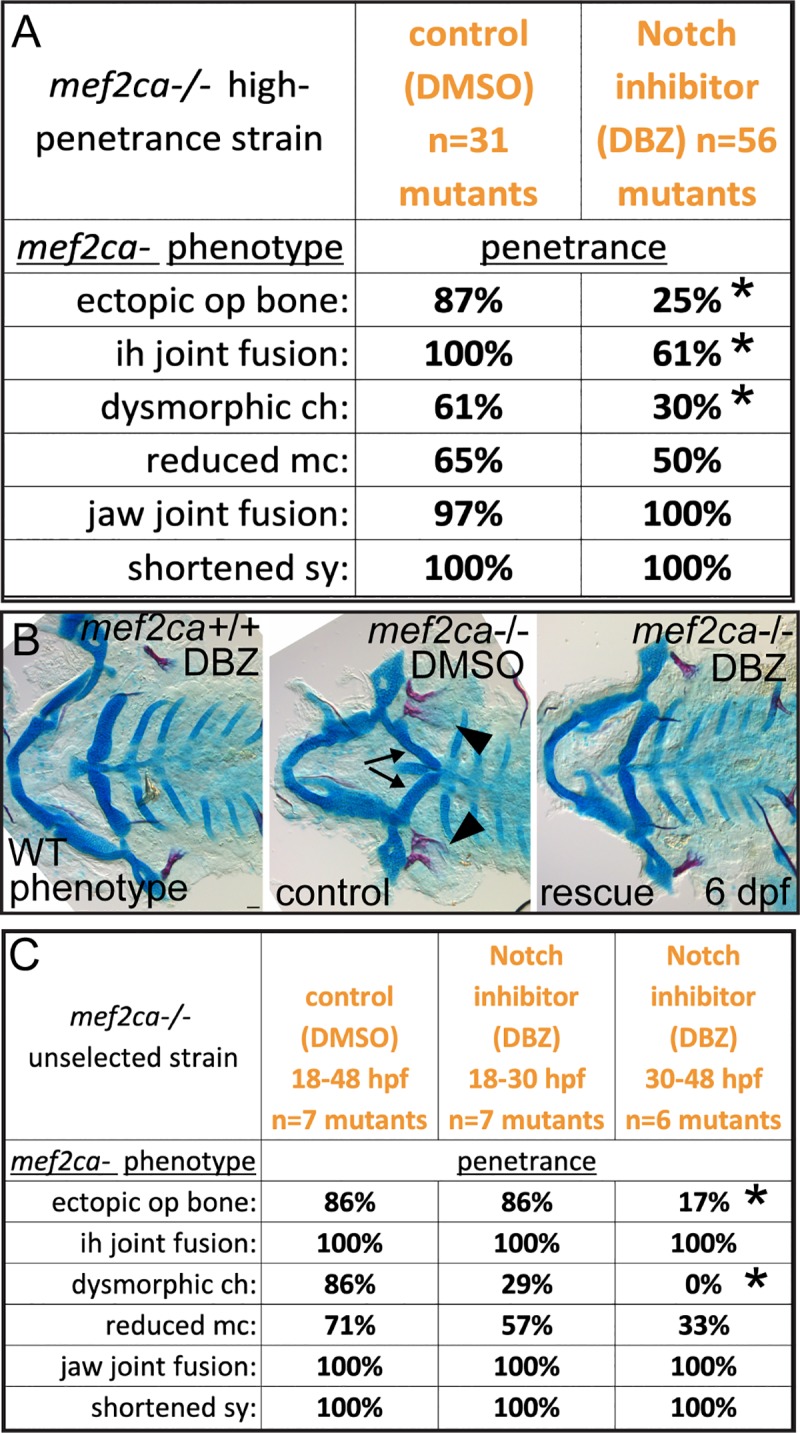
Pharmacological Notch inhibition decreases the penetrance of *mef2ca*-associated phenotypes without producing a skeletal phenotype in genetic wild types. (A) Animals heterozygous for *mef2ca* from the high-penetrance strain were intercrossed and offspring treated with Notch inhibitor (DBZ) or vehicle control (DMSO) from 18–48 hpf. Treated animals were fixed and stained with Alcian Blue (cartilage) and Alizarin Red (bone) at 6 dpf. Genotyped skeletal preparations were scored for penetrance of *mef2ca*-associated phenotypes. Asterisks indicate significance at alpha = 0.05 Fisher’s exact test compared with DMSO. (B) Imaged flat mounts of dissected skeletons from stained, genotyped animals treated with DMSO or DBZ were imaged. Arrows indicate dysmorphic ch and arrowheads mark ectopic bone. Scale bar: 50 μm. (C) Animals heterozygous for *mef2ca* from an unselected strain were intercrossed and offspring treated with either DMSO from 18–48 hpf, or Notch inhibitor (DBZ) from 18–30, or DBZ from 30–48 hpf. Treated animals were fixed and stained with Alcian Blue (cartilage) and Alizarin Red (bone) at 6 dpf. Genotyped skeletal preparations were scored for penetrance of *mef2ca*-associated phenotypes. Asterisks indicate significance at alpha = 0.05 Fisher’s exact test compared with DMSO.

The *mef2ca* gene functions in a genetic network downstream of Edn1 signaling. It has been previously shown that Edn1 signaling is opposed by the Jag/N signaling pathway, and that loss of Jag/N signaling can rescue loss of Edn1 signaling [29, 30]. Therefore, we hypothesized that alterations in Jag/N activity between strains might underlie differential *mef2ca* mutant penetrance. To directly test if Notch signaling functionally participates in *mef2ca* mutant penetrance, we used the characterized Notch signaling inhibitor dibenzazepine (DBZ) [53, 54]. DBZ is a gamma-secretase inhibitor that blocks processing of the Notch receptor into its active form [55]. Administering 10 μM of DBZ during zebrafish craniofacial development phenocopies mutation of the Notch ligand *jag1b* [30]. In our laboratory, we observe *jag1b* mutant phenocopy at 1 μM, and further reducing DBZ to 0.3 μM (administered 18–48 hpf to high-penetrance animals) significantly decreases penetrance of several *mef2ca* mutant craniofacial phenotypes (Fig 8A and 8B). These treatment conditions caused no detectable developmental phenotypes in genetic wild types. This finding shows that pharmacologically blocking the Notch pathway partially mimics the effects of the natural modifiers in our system, modifying penetrance without producing a phenotype in isolation. To determine the critical period when Notch signaling affects the *mef2ca* mutant phenotype, we repeated the experiment this time administering DBZ during two independent time windows, 18–30 hpf and 30–48 hpf. We found that inhibiting Notch signaling during the early time window does not significantly change the penetrance of any *mef2ca*-associated phenotypes. Conversely, inhibiting Notch signaling during the later time window significantly decreased the penetrance of the ectopic bone and ceratohyal phenotypes (Fig 8C). These data are congruent with our *dlx5a* expression experiment demonstrating that early defects in *dlx5a* recover later in development in low-penetrance *mef2ca* mutants.

**Fig 9:**
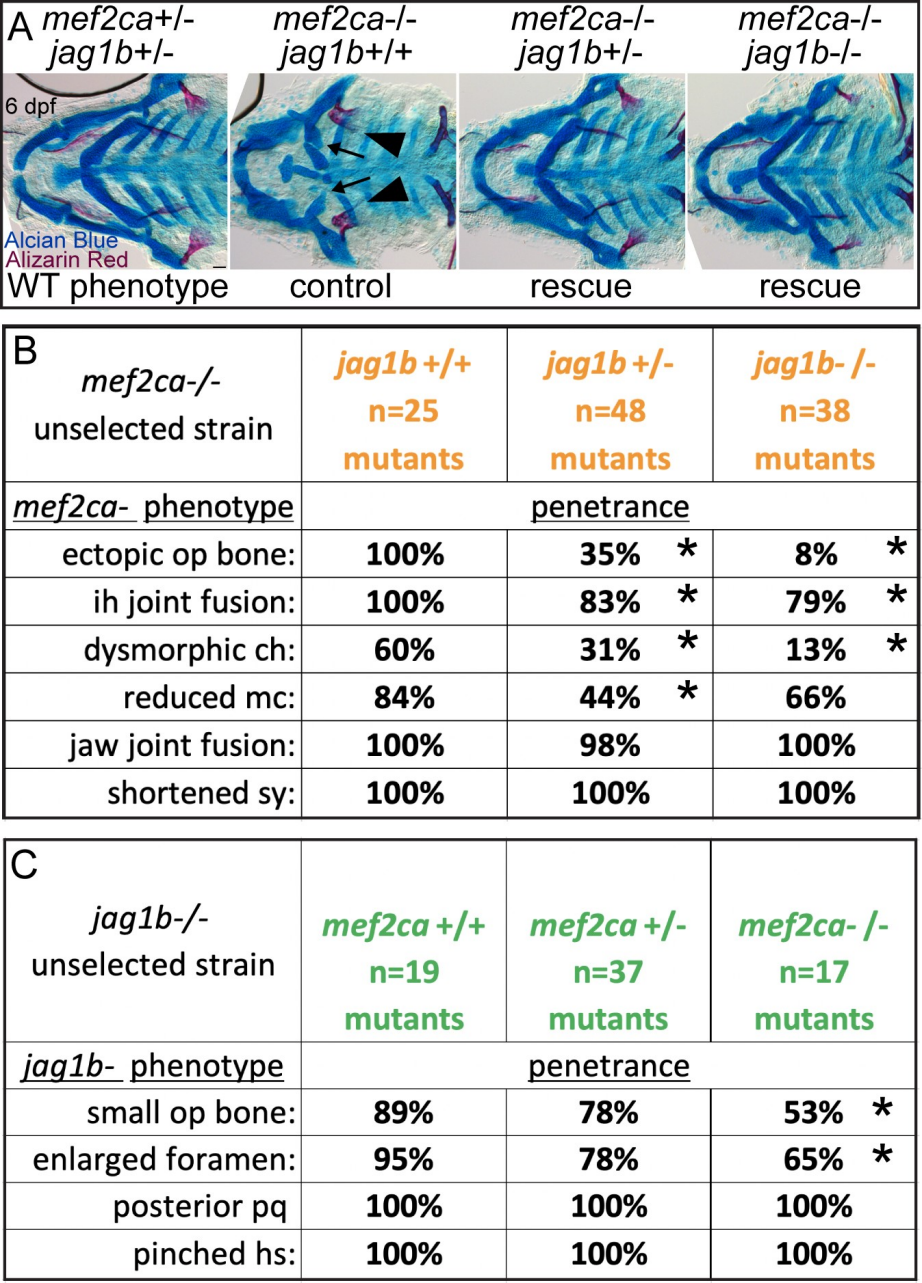
Loss of *jag1b* function decreases the penetrance of *mef2ca*-associated phenotypes. (A) Animals double heterozygous for *mef2ca* and *jag1b* were intercrossed, raised to 6 dpf and fixed and stained with Alcian Blue (cartilage) and Alizarin Red (bone). Genotyped animals were flat mounted and imaged. Removing functional copies of *jag1b* decreased *mef2ca* mutant penetrance. Scale bar: 50 μm. (B) Genotyped *mef2ca;jag1b* skeletal preparations were scored for penetrance of *mef2ca*-associated phenotypes. Asterisks indicate significance at alpha = 0.05 Fisher’s exact test compared with *jag1b+/+*. (C) Genotyped *mef2ca;jag1b* skeletal preparations were scored for penetrance of *jag1b*-associated phenotypes. Asterisks indicate significance at alpha = 0.05 Fisher’s exact test compared with *mef2ca+/+*.

We cannot be certain that this inhibitor only affects Notch signaling so we used a complementary genetic approach by crossing the loss of function *jag1b*^*b1105*^ mutant allele [29] into the high-penetrance strain. As predicted, removing a single functional copy of *jag1b* significantly decreased penetrance of most *mef2ca* mutant phenotypes, and penetrance is further decreased in the genetic doubly homozygous mutants (Fig 9A and 9B). It is interesting that experimentally disabling Jag/N does not rescue the jaw-joint fusion phenotype, whereas selective breeding does. These data suggest that changes to Jag/N might be primarily affecting the *mef2ca* phenotype in second pharyngeal arch-derived structures, while additional factors likely affect penetrance of *mef2ca*-associated phenotypes in first-arch derived structures. We also observe that some of the *jag1b* mutant-associated phenotypes are rescued by removing copies of *mef2ca* (Fig 9C) strengthening the model that *mef2ca* and Notch signaling oppose each other. Blocking Notch signaling partially rescues the, likely null, *mef2ca*^*b1086*^ mutation. Therefore, the molecular interaction is not likely to be through activated Notch directly inhibiting Mef2ca protein transcriptional activity as shown in cell culture overexpression [32, 33]. Instead, Notch signaling likely opposes *mef2ca* downstream of *mef2ca* transcriptional activity.

### *mef2ca* represses expression of the Notch ligand encoding gene *jag1b* equally in both strains late in pharyngeal arch patterning

**Fig 10:**
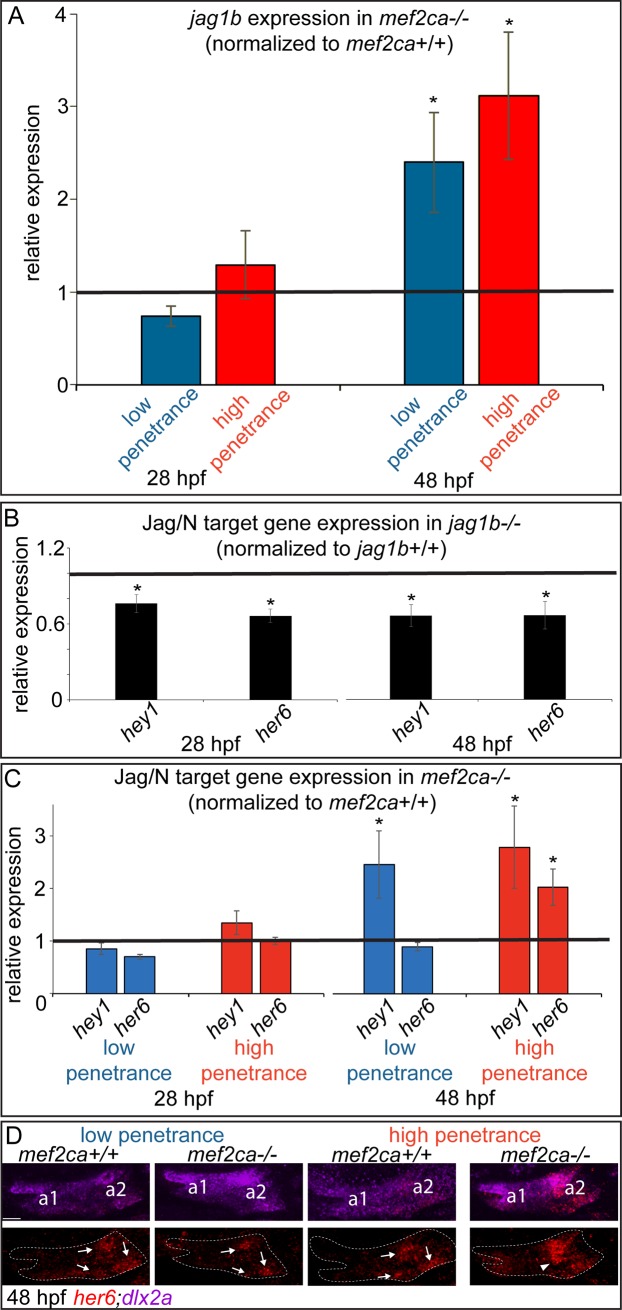
*jag1b* is similarly regulated between low- and high-penetrance strains, but the *jag1b* transcriptional target *her6* exhibits strain-specific regulation. (A) Head expression of *jag1b* was quantified by RT-qPCR in full-sibling *mef2ca* mutant and wild-type embryos from low- and high-penetrance strains at 28 and 48 hpf. Expression of *jag1b* in *mef2ca* mutants was normalized to wild-type sibling expression levels (black line). Asterisks mark expression levels significantly different in *mef2ca* mutants compared with *mef2ca* wild types (T-test at alpha = 0.05), error bars are standard deviation. (B) Head expression of the Jag/N transcriptional targets *hey1* and *her6* was quantified by RT-qPCR in full-sibling *jag1b* mutant and wild-type embryos at 28 and 48 hpf. Expression of each target in *jag1b* mutants was normalized to wild-type sibling expression levels (black line). Asterisks mark genes with expression levels significantly different in *jag1b* mutants compared with *jag1b* wild types (T-test at alpha = 0.05). Error bars are standard deviation. (C) Head expression of the Jag/N transcriptional targets *hey1* and *her6* was quantified by RT-qPCR in full-sibling *mef2ca* mutant and wild-type embryos from low- and high-penetrance strains at 28 and 48 hpf. Expression of each gene in *mef2ca* mutants was normalized to wild-type sibling expression levels (black line). Asterisks mark genes with expression levels significantly different in *mef2ca* mutants compared with *mef2ca* wild types (T-test at alpha = 0.05). Error bars are standard deviation. (D) Spatial expression of *her6* was determined by in situ hybridization. *her6* is expressed in three discrete patches in pharyngeal arch 2 (arrows) in low-penetrance wild types, low-penetrance mutants and high-penetrance wild types. In only high-penetrance *mef2ca* mutants *her6* expands ventrally (arrowhead). *dlx2a* expression was used to delineate pharyngeal arches one (a1) and two (a2) (dashed outlines). In the high penetrance strain, *her6* was expanded in 0/7 homozygous wild types and 7/8 homozygous mutants. In the low-penetrance strain, *her6* was expanded in 0/3 homozygous wild types and 0/5 homozygous mutants. Scale bar: 30 μm.

Our functional data indicate that experimental manipulations in Notch signaling can modify *mef2ca* mutant penetrance. Therefore, we hypothesized that changes in Notch signaling might underlie the differences between low- and high-penetrance selectively bred strains. To test this hypothesis, we examined expression of the Notch ligand *jag1b* in both the low- and high-penetrance strains. Surprisingly, we see no significant difference in *jag1b* expression between strains at either early or late time points (Fig 10A). At 28 hpf *jag1b* expression is similarly unaffected by the *mef2ca* mutation in both strains and at 48 hpf *jag1b* expression is similarly significantly upregulated by the *mef2ca* mutation in both strains. These data are consistent with the model that Edn1 signaling acting through *mef2ca* represses *jag1b* expression and thus antagonizes Notch signaling. However, these data also indicate that differences in *jag1b* mRNA expression do not account for differences in *mef2ca* mutant penetrance between strains. We have not ruled out that strain-specific levels of Jag1b protein or posttranslational modifications, known to affect Notch ligand activity [56], might activate Notch downstream targets differentially in the two strains.

### The Notch transcriptional target gene *her6* is differentially regulated between low- and high-penetrance strains

Given the convincing evidence that Jag/N signaling functionally modifies *mef2ca* mutant penetrance, but *jag1b* expression dynamics are essentially identical between our low- and high-penetrance strains, we next investigated canonical downstream Notch targets. The *hes-related family bHLH transcription factor with YRPW motif 1* (*hey1*) and *hairy-related 6* (*her6*) genes are canonical Notch targets expressed in the zebrafish pharyngeal arches [29, 49, 57, 58]. We find that expression of these Notch targets is dependent upon *jag1b* function (Fig 10B). Unlike *jag1b*, which behaves similarly between low- and high penetrance *mef2ca* strains, we find that *her6*, but not *hey1*, displays strain-specific expression differences. Specifically, *her6* is up regulated in *mef2ca* mutants from the high-penetrance strain compared to wild types at 48 hpf (Fig 10C), similar to *jag1b* (Fig 10A). In contrast, *her6* is not upregulated in low-penetrance *mef2ca* mutants compared with low-penetrance wild-type controls at this stage. The other Jag/N target *hey1* does not show strain-specific expression; it is upregulated in *mef2ca* mutants in both low- and high-penetrance strains. To determine if *her6* is specifically upregulated in the ventral arch cells affected in *mef2ca* mutants from the high-penetrance strain, but not the ventral cells that are unaffected in *mef2ca* mutants from the low-penetrance strain, we performed in situ hybridization (Fig 10D). In support of the qPCR data, we find that *her6* is not upregulated in low-penetrance mutants compared with wild types. In contrast, *her6* is ventrally expanded in *mef2ca* mutants from the high-penetrance strain compared with high-penetrance wild types. Thus, *her6* is only upregulated in the ventral domain of high- but not low-penetrance *mef2ca* mutants. To test if the observed upregulation of *her6* in high-penetrance mutants might be sufficient to increase penetrance, we overexpressed *her6* in unselected *mef2ca* mutants. In these experiments, we discovered that injecting *her6* mRNA significantly increased ectopic bone penetrance from 18% n = 44 in buffer injected controls to 41% n = 44 in *her6* overexpression conditions (p-value = 0.02 Fisher’s exact), phenocopying the high-penetrance mutants.

These data suggest that this specific node in the opposing Jag/N signaling network, *jag1b* driving *her6* expression, is disabled in only the low-penetrance strain. Thus, strain-specific Notch signaling circuitry likely contributes to low penetrance in our selectively-bred strain.

### Epistasis orders modifiers in a genetic pathway

**Fig 11:**
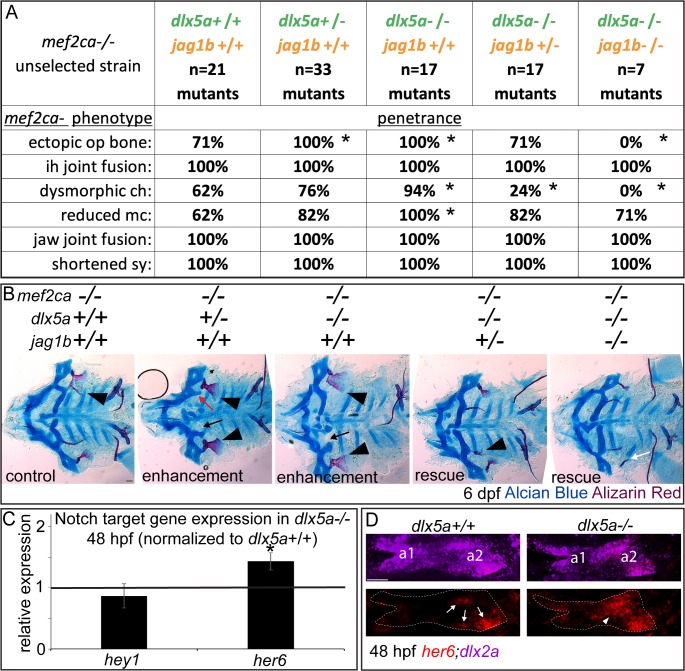
Triple mutants reveal *mef2ca* modifier epistasis. (A) Animals triple heterozygous for *mef2ca*, *dlx5a* and *jag1b* were intercrossed, offspring were raised to 6 dpf and fixed and stained with Alcian Blue (cartilage) and Alizarin Red (bone). Genotyped skeletal preparations were scored for penetrance of *mef2ca*-associated phenotypes. Removing functional copies of *jag1b* from *mef2ca;dlx5a* double mutants decreases penetrance of *mef2ca*-associated phenotypes. Asterisks indicate significance at alpha = 0.05 Fisher’s exact test compared with *mef2ca-/-;dlx5a+/+;jag1b+/+*. (B) Genotyped *mef2ca;dlx5a;jag1b* skeletal preparations were flat mounted and imaged. *mef2ca* mutant associated phenotypes are indicated including ectopic bone (arrowheads), dysmorphic ch (arrows) and ch-to-hm transformation (red arrow). Stick-like opercle bone is indicated with a white arrow. Scale bar: 50 μm. (C) Head expression of Notch target genes was quantified by RT-qPCR in full-sibling *dlx5a* mutant and wild-type embryos at 48 hpf. Expression in *dlx5a* mutants was normalized to wild-type sibling expression levels (black line). Asterisks mark genes with expression levels significantly different in *dlx5a* mutants compared with *dlx5a* wild types (T-test at alpha = 0.05). Error bars are standard deviation. (D) Spatial *her6* expression was monitored by in situ hybridization in wild types and *dlx5a* mutants. In wild-type embryos *her6* is expressed in three discrete patches in arch 2 (arrows). In *dlx5a* homozygous mutants, *her6* expands ventrally resulting in a larger, more continuous expression domain (arrowhead). *dlx2a* expression was used to delineate pharyngeal arches one (a1) and two (a2) (dashed outlines). *her6* was expanded in 0/4 homozygous wild types and 3/3 homozygous mutants Scale bar: 30 μm.

We next ordered the genetic modifiers we discovered to have strain-specific expression differences, *dlx5a* and Notch signaling, within a genetic pathway. Because the *dlx5a* mutation increases *mef2ca* mutant penetrance and the *jag1b* mutation decreases *mef2ca* mutant penetrance, these genes exhibit opposite mutant phenotypes in this context. To determine the order in which these modifiers function in a genetic pathway, we used genetic epistasis [59]. By intercrossing *mef2ca*;*dlx5a*;*jag1b* triple heterozygotes, we generated a full-sibling family with all allelic combinations of these interacting genes. For simplicity, we only consider *mef2ca* homozygous mutants in these analyses in order to focus on the modifier epistasis. We found that removing functional copies of *dlx5a* in *mef2ca* mutants increased the penetrance of *mef2ca-*associated phenotypes (Fig 11A and 11B), consistent with our earlier experiments (Fig 7). Further, removing functional copies of *jag1b* from *mef2ca;dlx5a* homozygous double mutants reduced penetrance. Thus, even when *mef2ca* and *dlx*5a are doubly homozygous mutant, the *jag1b* mutant still rescues the *mef2ca* mutant-associated phenotypes. Therefore, we consider the modifier function of *jag1b* to be epistatic to the modifier function of *dlx5a*. Moreover, because the triple-mutant opercle bones had reduced, stick-like shapes (Fig 11B), a phenotype associated with *jag1b* single mutants [29, 30], we consider *jag1b* to be epistatic to both *mef2ca* and *dlx5a*.

One hypothesis consistent with these epistasis results is that *jag1b* acts positively and *dlx5a* acts negatively on a shared downstream target. To test this hypothesis, we examined the canonical Notch targets positively regulated by *jag1b* (Fig 10B) to determine if they were negatively regulated by *dlx5a*. Consistent with our epistasis interpretation, we find that *her6*, but not *hey1* is significantly upregulated in *dlx5a* mutants (Fig 11C). To test if *her6* is upregulated in ventral cells in the arches of *dlx5a* mutants we performed in situ hybridization (Fig 11D). Consistent with the qPCR data, and similar to the *her6* expression data in high-penetrance *mef2ca* mutants (Fig 10C and 10D), *her6* expression is ventrally expanded in *dlx5a* mutants compared with wild type.

These experiments reveal part of the wild-type genetic circuit downstream of *mef2ca* and are all consistent with our model that *jag1b*-dependent transcription of *her6* is disabled in the low-penetrance strain to tune the opposing Notch pathway and rescue the *mef2ca* mutation.

## Discussion

Incomplete penetrance complicates both our understanding of developmental genetics and progress toward personalized medicine. Moreover, if we can understand why phenotypes only sometimes manifest in mutants we may someday capitalize on natural mechanisms of genetic resilience for human therapies. Here, we took an unconventional approach to understand incomplete penetrance by starting with a mutant that variably displayed a phenotype and driving penetrance downward and upward so that strains with differential penetrance could be carefully compared. This approach revealed one mechanism allowing organisms to overcome a deleterious mutation, the tuning of an opposing genetic circuit. While we find that manipulating Jag/N does partially phenocopy our selective breeding, it does not rescue the jaw-joint fusion phenotype. It would be interesting to learn if performing a new selective breeding experiment for jaw-joint penetrance would uncover a different set of modifiers. These data suggest that while tuning the opposing Jag/Notch pathway is a key factor in the rescue in the low-penetrance strain, other pathways like BMP are also likely at play. Therefore, we conclude that the ability of organisms to overcome a deleterious mutation is likely due to changes in multiple opposing pathways. Because opposing circuits are common in development, it is possible that tuning opposing circuits is a widespread mechanism underlying incomplete penetrance in many systems.

### Penetrance inheritance fits the liability-threshold model

**Fig 12:**
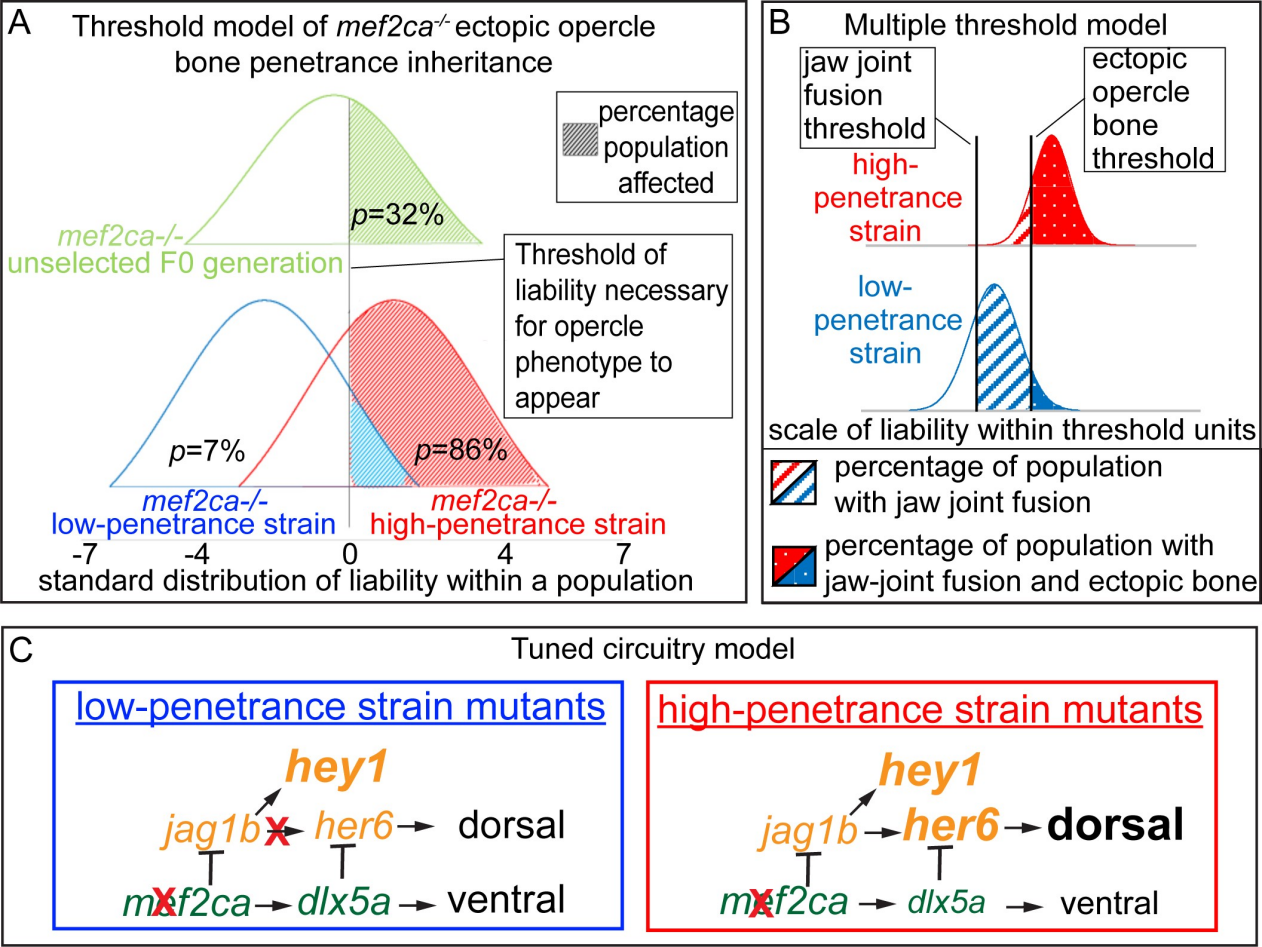
Liability-threshold model and tuned circuitry model for *mef2ca* heritable variable penetrance. (A) The liability-threshold model illustrates the inheritance of ectopic bone penetrance in *mef2ca* mutants following selective breeding. The difference between the mean of the population and the threshold is defined as *x*. *x*_F0-op_ = 0.468, *x*_high-op_ = -1.080 and *x*_low-op_ = 1.918. (B) The multiple threshold model illustrates how the same modifiers can shape liability and penetrance of different *mef2ca* mutant phenotypes to different extents. *x*_high-jaw joint_ = -4, *x*_low-jaw joint_ = -.806 *x*_high-op_ = -1.08 *x*_low-op_ = 1.476. (C) Model for how tuning down of Jag/N-driven transcription of *her6* can result in *mef2ca* low penetrance. This genetic circuitry downstream of *mef2ca* is consistent with mutant, genetic epistasis, and gene expression analyses in this study.

Our penetrance inheritance pedigrees indicate that the inheritance is not due to Mendelian inheritance of a single locus. Rather, our data fit the liability-threshold model of inheritance (Fig 12A). This model predicts that multiple genes control liability, which is a normally distributed continuous trait in a population. Once an individual passes a threshold of liability, they exhibit the trait. Thus, in this model, a discontinuous trait has underlying continuity. We propose that ectopic bone is a discontinuous trait with underlying continuity. Penetrance is not controlled by a single modifier locus, but rather is likely controlled by relatively few loci as low and high penetrance is quickly rederived after outcrossing (Fig 1). To apply the threshold trait model to our data, we calculated the mean liability of populations using the incidence of ectopic bone before and after selection (Fig 12A). We find that the standard deviation of liability following selection differs between low- and high-penetrance strains by 2.6 standard deviations of liability supporting the threshold model. For reference, a meaningful difference in liability is 0.8 and above [60].

Our phenotype scoring revealed that the various *mef2ca*-asssociated phenotypes were affected to different extents by selection on just the ectopic bone phenotype (Fig 2). This observation strengthens the liability-threshold model of heritable penetrance if we consider the multiple threshold model [60]. In this model, the same liability applies to different thresholds. Thus, appearance of different phenotypes requires different levels of the same liability, explaining why the same selection paradigm might affect different phenotypes to different extents. To visualize our data in the context of the multiple threshold model, we plotted our phenotype data for two *mef2ca*-associated phenotypes (Fig 12B). This model predicts that phenotypes like jaw-joint fusions would be observed more frequently in Edn1 pathway loss of function mutants, occurring when other *mef2ca*-associated phenotypes like ectopic bone are not present. This prediction is supported by the finding that weak loss of Edn1 pathway conditions like *furina* mutations or low dose *edn1* morpholino knockdown show jaw-joint fusions without ectopic bone, suggesting that jaw-joint fusions are particularly sensitive to partial loss of Edn1 signaling [31].

We find that genetic and pharmacological manipulations can shift the liability distribution and phenocopy the changes that emerge during selective breeding. In some cases, genetic manipulations might shift the liability beyond what is thus far achieved by selective breeding. For example, the duplicated foramen phenotype (Figs 7A and 11B) is present in 4/101 (4%) of *mef2ca*^-/-^;*dlx5a*^+/-^ mutants but is not yet observed in *mef2ca* single mutants, even in the high-penetrance strain. Thus, we predict that through continued selective breeding this duplicated foramen phenotype might emerge in the high-penetrance strain as we push the liability further through selection, matching the *dlx5a* loss of function condition. While we do not know if an ectopic nerve fiber is present in these duplicated foramina, this dramatic transformation raises the idea that a broad-scale identity transformation occurs in these conditions including the ventral cells adopting an intrinsic dorsal propensity to form a foramen. There is a precedent for dorsally transformed ventral cells to adopt this intrinsic dorsal propensity to form a foramen; mouse *Ednra* mutants form a duplicated maxilla complete with foramina [61]. Nearly perfect transformations like these are not observed in the zebrafish strong loss of Endothelin signaling condition arising from the *edn1*^*tf216b*^ mutation, likely because these *edn1* mutants also present a ventral hypoplasia phenotype [62], which might preclude perfect transformations because there simply aren’t enough cells.

### Selective breeding can alter allele behavior

Another prediction of the liability-threshold model is that the liability can be shifted to such an extreme that eventually the phenotypes normally associated with *mef2ca* homozygous mutants begin to appear in *mef2ca* heterozygotes, which we observe in our system. We previously pointed out similarities between our selective breeding experiment and those performed by Waddington which demonstrated the phenomenon of genetic assimilation of an acquired character [20, 63]. The genetic assimilation phenomenon can be explained with the liability-threshold model [60]. Waddington applied selective breeding to enrich for phenotypes that appear as a result of an environmental stimulus until the phenotype appeared without stimulation. We applied selective breeding to enrich for phenotypes that appear as a result of a homozygous mutation until the phenotypes appeared without homozygosity. In both cases the distribution of the liability within the population was shifted with regard to the threshold by selective breeding. While it was a surprise that a fully recessive allele could be converted to a partial dominant by selective breeding, the original description of the *mef2ca* mutant allele reported a heterozygous *mef2ca* phenotype when *edn1* is also heterozygous [24]. Moreover, in humans heterozygous *MEF2C* mutations produce craniofacial phenotypes. Therefore, in some sensitized contexts *mef2ca* mutant alleles can be dominant. Furthermore, Waddington’s genetic assimilation model might predict that in future generations of selective breeding gain- or loss-of-Notch phenotypes might appear as the opposing circuit is further tuned. In a Waddington-like fashion, after more generations of selective breeding for high penetrance, it’s possible that the background may become so severe that *mef2ca* mutant-associated phenotypes appear in wild-type animals with two fully-functional copies of *mef2ca*. Together with the discovery that selective breeding can change the essentiality of *mef2ca* (Fig 4A), our study demonstrates the fluidity of allele behavior. Moreover, the findings presented here emphasize the importance of understanding and controlling for genetic background influences on phenotypes.

### Genetic circuitry tuning emerges through selection

We find that one mechanism of overcoming a deleterious mutation that can arise from artificially selecting for mutant penetrance is the emergence of a newly-tuned genetic network. Specifically, selective breeding for low penetrance of a dorsalizing mutation disabled a specific node in the pathway specifying dorsal identity. Thus, restoring the balance between dorsal and ventral specifying gene expression (Fig 12C). These results suggest that transcriptional networks may have a high degree of plasticity and that similar mechanisms might be at work in natural selection. In fact, we propose that selective pressure is likely at work during normal stock husbandry. In this model, any subtle heterozygous phenotypes might be quickly selected against inadvertently by selecting healthy, fast-growing individuals to propagate a line during normal stock maintenance. In this regard, slight differences in genetic circuitry might be at work in various different mutant strains within a zebrafish colony.

We find timing is an important factor in uncovering circuitry differences. For example, low- and high-penetrance strains behave similarly early during craniofacial patterning, but the strain-specific circuitry emerges only later. Consistently, we find that inhibiting Notch signaling late, but not early, in craniofacial development rescues *mef2ca* mutant phenotypes. These results indicate that the neural crest cells are initially equally disrupted by the mutation, but in the low-penetrance strain the tuned Jag/Notch signaling pathway allows the low-penetrance mutants to later overcome the mutation. Similar phenomena occur in unselected strains, where the *mef2ca* transcriptional target *hand2* is initially downregulated in mutants then recovers over time [24]. This phenomenon is likely due to changes in BMP signaling during development [57, 64]. Similarly, in *furina* mutants *dlx5a* expression is initially disrupted but then later recovers. This *dlx5a* recovery phenomenon is not observed in *edn1* mutants indicating only some craniofacial mutants can recover over time [31]. Other mutations affecting neural crest cells, like those in *prickle1*, also exhibit early phenotypes that are later overcome [65]. Therefore, we propose that the ability of some mutants to overcome a deleterious mutation truly is a form of resilience, defined as the ability to recover from or adjust to misfortune or change over time [66].

## Methods

### Zebrafish strains and husbandry

All fish were maintained and staged according to established protocols [67, 68]. Selective breeding was performed as previously described [20, 37]. The three previously described zebrafish *mef2ca* mutant alleles are all recessive and homozygous lethal in unselected backgrounds. The *mef2ca*^*b1086*^ mutant allele we exclusively use in this study and the KASP genotyping (LGC) protocol for this allele using a StepOnePlus Real-Time PCR System (Applied Biosystems) has been previously described [20, 24, 37]. The *jag1b*^*b1105*^ zebrafish line and genotyping protocol has been previously described [29]. The *dlx5a*^*j1073Gt*^ line has been previously described [47]. For genotyping *dlx5a*, heterozygotes and homozygous mutants were discriminated from wild types based on EGFP fluorescence. Among EGFP positive animals, heterozygotes were discriminated from homozygous mutants by PCR amplifying a wild type-specific amplicon with primers CGTAACAGCGCAATTTAGGA and GTTGTGATTGCACTCTGTTATATGT which flank the transgene integration site.

### Pharmacological treatments

Nonsense-mediated decay was inhibited as previously described [43]. Briefly, offspring from intercrosses from low-penetrance *mef2ca* heterozygotes were treated with 10 μM NMDi14 (Calbiochem) or DMSO from 18–42 hpf. At 6 dpf animals were stained for bone and cartilage and genotyped *mef2ca* homozygous mutants were scored for *mef2ca* associated phenotypes. No gross alterations were observed in the DMSO- or drug-treated wild types or heterozygotes. Notch signaling was inhibited similar to previous experiments [30]. Offspring from intercrosses from high-penetrance *mef2ca* heterozygotes were treated with 0.3 μM DBZ (Tocris) or DMSO control from 18–48 hpf, 18–30 hpf, or 30–48 hpf.

### Phenotype scoring

Genotyped Alcian Blue/Alizarin red skeletons were scored for penetrance, the proportion of mutant animals that displayed a particular phenotype that differed from the wild type. We did not score for expressivity or severity of phenotype. Penetrances were compared by Fisher’s exact test to determine significance. All scoring data and exact p values are reported in supplementary data table (S1 Data). In the interest of strong rigor and reproducibility, more subjective phenotypes like reduced Meckel’s cartilage and dysmorphic ceratohyal were scored by three blinded observers to determine if penetrance could be reproducibly scored by different individuals. Specifically, nine animals from the low-penetrance strain and eight animals from the high-penetrance strain were pooled and determined by an observer to have 10/17 animals with dysmorphic ceratohyal cartilage and 9/17 with reduced Meckel’s cartilage. Then three observers blind to genotype, strain, and the initial scoring scored the same pool of animals. All three agreed with the number of animals in each phenotypic class. This experiment suggests that phenotype penetrance can be reproducibly identified by different observers.

### Tissue labeling

For staining cartilage and bone, fixed animals were stained with Alcian Blue and Alizarin Red as described previously [69]. For staining muscle and bone, live larvae were stained with 0.005% Alizarin Red in embryo media buffered with HEPES before fixation in 4% PFA with PBS overnight. Fixed larvae were permeabilized with 1% TritonX for 6 hours then stained with 1:100 fluorescently conjugated phalloidin overnight at 4C.

### RT-qPCR and RT-PCR

For RT-qPCR, live individual 28 or 48 hpf embryos from each strain had their heads removed. Decapitated bodies were genotyped to identify homozygous wild-type and homozygous *mef2ca*^*b1086*^, *jag1b*^*b1105*^ *or dlx5a*^*j1073Gt*^ mutant individuals. Heads from 5–6 identified homozygous wild types and mutants were pooled and total RNA was extracted with TRI Reagent. cDNA was prepared with Superscript III from Invitrogen. qPCR experiments utilized a Real-Time PCR StepOnePlus system from Applied Bio system and sybr green. A standard curve was generated from serially diluted (1:2:10) cDNA pools and primers with a slope of -3.3 +- 0.3 were accepted. The relative quantity of target cDNA was calculated using Applied Biosystem StepOne V.2.0 software and the comparative Ct method [70]. After surveying the expression of many housekeeping genes at multiple stages [71] we determined that *rps18* was the most consistent across stages, genotypes and strains. Target gene expression in all experiments was normalized to *rps18*. Reactions were performed in technical triplicate and the results represent two to six biological replicates. The following primers were used: *rps18* FW, 5’-CTGAACAGACAGAAGGACATAA-3’ and *rps18* REV 5’-AGCCTCTCCAGATCTTCTC-3’, *mef2ca* FW, 5’-GTCCAGAATCCGAGGACAAATA-3’ and *mef2ca* REV 5’-GAGACAGGCATGTCGTAGTTAG-3’, *mef2cb* FW, 5’-AGTACGCCAGCACAGATA-3’ and *mef2cb* REV 5’-AGCCATTTAGACCCTTCTTTC -3’, *mef2aa* FW, 5’-CCAGAGAGCAGAACCAACTC-3’ and *mef2aa* REV 5’-GTCCATGAGGGGACTGTGAC-3’, *mef2ab* FW, 5’-AACCTCACGAGAGCAGAACC-3’ and *mef2ab* REV 5’-AGGACATATGAGGCGTCTGG-3’, *mef2b* FW, 5’-CCGATATGGACAAAGTGCTG-3’ and *mef2b* REV 5’-CCAATCCCAATCCTTTCCTT-3’, *mef2d* FW, 5’-TTCCAGTATGCCAGCACTGA-3’ and *mef2d* REV 5’-CGAATCACGGTGCTCTTTCT-3’, *dlx5a* FW, 5’-CGTATCACCAATACGCAGGA-3’ and *dlx5a* REV 5’-AGTAAATGGTTCGGGGCTTC-3’, *jag1b* FW, 5’-CGCTAAGTCATGCCACAA -3’ and *jag1b* REV 5’-CACTGACCCTTACAGTCATTTA-3’, *hey1* FW, 5’-CTCCATCCACAACCTCTCAA-3’ and *hey1* REV 5’- CGCAGCTCAGATAAACTGTTATT-3’, *her6* FW, 5’-AACGAAAGCTTGGGTCAG-3’ and *her6* REV 5’-ACTGTCATCTCCAGGATGT-3’. For RT-PCR to test for mutant exon splicing, heads from five genotyped homozygous wild types and *mef2ca*^*b1086*^ mutants from each strain were pooled and total RNA was extracted with TRI Reagent. cDNA was prepared with Superscript III from Invitrogen. PCR was performed with primers spanning the mutant exon resulting in a predicted full-length product of 381 bp and a spliced product of 177 bp. The primers were: F1 5’-CAGAAGTCATGGGGAGGAAA-3’ and R1 5’-GGTCGATGTCCTCGTTGATT-3’. For maternal deposition, wild type RNA was extracted from pooled embryos using Direct-zol extraction columns and then DNase I treated. First-Strand cDNA synthesis was performed using Superscript III reverse transcriptase (RT +) or water as a RT negative control (RT -). PCR was performed with GOTAQ Green supermix on samples using primers spanning a splice junction: F2 5’-GAAGAAGGCCTACGAGCTGA-3’ and R2 5’-AGGTCGATGTCCTCGTTGAT-3’. All qPCR numerical data and statistical analyses are reported in supplementary data table (S1 Data).

### RNA in situ hybridization

Whole-mount in situ hybridization with fluorescence detection was performed as previously described [72]. The *dlx2a* [73] and *her6* [49] probes have been previously described.

### Immunostaining

28hpf *mef2ca*^*b1086*^ wild-type and mutant embryos were fixed overnight in BT fix (4%PFA, 4% sucrose, 0.12 mM CaCl2 in PBS) at 4°C. Embryos were permeabilized in 150 mM Tris-HCL pH 9.0 at 70°C for 15 minutes and acetone for 20 minutes at -20°C, then washed 6 times in PBST. Blocking solution (1%BSA, 2%Serum, 1%DMSO, 0.1% Tween 20 in PBS) was applied for 2 hours at room temperature followed by rabbit anti-MEF2C (Sparrow Biosciences) primary antibody (1:1000) overnight in blocking solution. Several rinses in washing solution (0.5% Triton X, 2% DMSO, 0.1% Sodium Azide) preceded detection with (1:1000) Alexa Fluor 568 goat anti-rabbit secondary antibody overnight in block.

### *her6* overexpression

We amplified the T7 promoter followed by the *her6* native Kozak sequence, the *her6* open reading frame and partial 3’UTR from cDNA prepared from 28 hpf wild-type embryos using primers F 5’-AGAGAAGATGCCTGCCGATA-3’ and R 5’-CGCTGAACAAAGAAAACAAGTG-3’. Amplified fragments were cloned using pCR-Blunt II-TOPO and *her6* clones were identified by restriction digest and Sanger sequencing. Using Phusion polymerase, a *her6* template was amplified from the cloned plasmid and RNA for injection was synthesized using the T7 mMessage Machine transcription kit. RNA was purified by lithium chloride precipitation. A final dose of 80 pg was injected into one-cell stage embryos produced from intercrosses of unselected *mef2ca* heterozygotes.

### Microscopy and image analysis

Alcian Blue/Alizarin Red stained skeletons were dissected and flat mounted for imaging on a Leica DMi8 inverted microscope equipped with a Leica DMC2900. Fluorescent images were captured using a Leica DMi8 equipped with an Andor Dragonfly 301 spinning disk confocal system. Images were assembled in Imaris and Photoshop with any adjustments applied linearly and equally to all panels.

### Threshold character calculations

The difference between the mean of the population and the threshold (*x*) was determined from the percent of population affected as previously described [60]. To compare difference in liability between low- and high-penetrance strains, we calculated the difference between the two means of the populations.

### Statistical analyses

For penetrance scores, we performed the Fisher’s exact test using GraphPad, and adjusted p-values for multiple comparisons using the False Discovery Rate (FDR) adjustment in R as well as the Hochberg procedure in R. For qPCR data, we performed t-tests followed by Tukey Honestly Significant Difference (HSD) tests to account for multiple comparisons in R. All raw data and exact p-values from the various statistical analyses are reported in the supplementary worksheet (S1 Data).

### Ethics statement

All of our work with zebrafish has been approved by the University of Colorado Institutional Animal Care and Use Committee (IACUC), Protocol # 00188. Animals were euthanized by hypothermic shock followed by 1.5% sodium hypochlorite.

## Supporting information

S1 Data Worksheet containing all raw numerical data and statistical analyses. For qPCR data, all C_T_ and mean 2^-ΔΔCT^ values are shown. Exact p-values from statistical significance tests (t-test and Tukey’s HSD) are shown. Bolded values are statistically significant. For penetrance data, all scoring data is presented. Exact p-values from statistical significance tests (Fisher’s exact and FDR adjustments) are shown.
(XLSX)
